# Twisted optical fibres as photonic topological insulators

**DOI:** 10.1038/s41566-026-01848-9

**Published:** 2026-02-20

**Authors:** Nathan Roberts, Brook Salter, Jack Binysh, Peter J. Mosley, Anton Souslov

**Affiliations:** 1 Department of Physics, University of Bath, Bath, UK; 2 Centre for Photonics, University of Bath, Bath, UK; 3 National Research Council of Canada, Ottawa, Ontario Canada; 4 Department of Physics, University of Ottawa, Ottawa, Ontario Canada; 5 Institute of Physics, Universiteit van Amsterdam, Amsterdam, the Netherlands; 6 TCM Group, Cavendish Laboratory, Cambridge, UK

**Keywords:** Fibre optics and optical communications, Metamaterials

## Abstract

The breaking and enforcing of symmetries is a crucial ingredient in designing topologically robust materials. In electronic and microwave systems, magnetic fields can break time-reversal symmetry to create Chern insulators. By contrast, at optical frequencies, natural materials cannot respond to magnetic fields, which presents a challenge for the scalable exploitation of topologically enhanced devices. Here we leverage the natural geometry of fibre to build a scalable photonic Chern insulator by twisting the fibre during fabrication. The twist inside optical fibre breaks an effective time-reversal symmetry and induces a pseudo-magnetic field, which we observe via photonic Landau levels. Unavoidably, this twist introduces a competing topology-destroying effect through a parabolic profile in the effective refractive index. Using simulations to guide experimental materials design, we discover the ‘Goldilocks’ regime where the real-space Chern invariant survives, guaranteeing topological protection against fabrication-induced disorder of any symmetry class.

## Abstract (web summary)

In this work, researchers build a scalable photonic Chern insulator by twisting a fibre during fabrication, breaking an effective time-reversal symmetry and inducing a pseudo-magnetic field. The team reveals a ‘Goldilocks’ regime that guarantees topological protection against fabrication-induced disorder of any symmetry class in the fibre cross-section.

## Main

Topological band engineering enables the creation of robust propagating states in a variety of media^1^. In electronic^2,3^ and microwave systems^4,5^, magnetic fields are routinely used to break time-reversal symmetry and create Chern insulators. These topological materials act as insulators in the bulk but display quantized modes at the boundary, which are robust against disorder and localization^1,6^. However, natural materials do not respond to magnetic fields at optical frequencies, creating a challenge for photonic Chern insulators.

Without breaking time-reversal symmetry, topology has previously been exploited through quantum spin-Hall^7–11^ and valley-Hall^12–15^ effects. Unlike Chern insulators, these systems are robust only against select types of disorder^16,17^, but topology still offers some protection, for example, to entangled quantum states^18,19^. An alternative approach to exploit non-trivial topology is to use time-varying metamaterials with a Floquet state^20–24^. However, the necessary fabrication processes, for example, direct-written photonic waveguides, leave these platforms limited in size, flexibility and scalability. Although optical fibre has been hypothesized to exploit topology on the largest scales^25–30^, previously fabricated topological fibre^31,32^ did not break time-reversal symmetry and was only topologically robust against sub-classes of disorder.

**Fig. 1:**
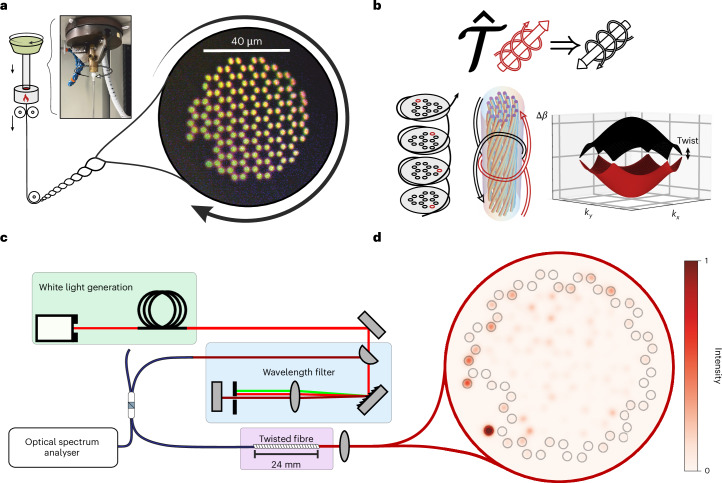
Twisted optical fibre overview. **a**, Diagrammatic explanation of the drawing process. We rotate the preform while feeding it into the furnace to induce a twisted structure. The fibre cross-section features a notch to demonstrate that a topological mode can be guided around an arbitrarily shaped edge. The inset photograph shows a preform being twisted and the micrograph shows the fabricated fibre cross-section with light coupled into the Ge-doped cores. **b**, Fibre twist schematic showing the breaking of the effective time-reversal symmetry $\widehat{{\mathcal{T}}}$ (equivalently, *z*-propagation symmetry) owing to the applied twist. The band structure shows Dirac points in an infinite untwisted system, which are gapped when an effective magnetic field is introduced. This infinite-lattice model captures the topology introduced through the vector potential, but misses the crucial effects of the periodicity-breaking scalar potential. **c**, Experimental set-up for observing the intensity profile of an excited edge. We use a supercontinuum-generating fibre as a source of white light, which we filter, and butt-couple light at a desired wavelength into our twisted fibre. **d**, Light has been injected into a single core on the edge of the fibre and propagated over a length of 24 mm. The injection core has been overexposed in this image to highlight the intensity distribution in the other excited cores and to show where light is initially injected (compare with Fig. 2). The edge cores are outlined for reference.

By including many germanium-doped cores inside the cross-section of a single optical fibre, we build a honeycomb lattice that supports collective supermodes. We engineer the structure of the supported supermodes to contain Dirac-point band crossings, and twist the fibre to open a gap characterized by a Chern topological invariant. We augment the usual stack-and-draw fabrication process by spinning the fibre to freeze-in a constant twist (Fig. 1a), which breaks propagation symmetry (Fig. 1b) and induces experimentally observable robust edge-localized modes (Fig. 1c–d). Importantly, this propagation symmetry is mathematically analogous to time-reversal symmetry in the Schrödinger equation, and we describe the twisted fibre as breaking an effective time-reversal symmetry. Spinning the fibre during fabrication presents a scalable way to twist all the cores simultaneously (to contrast with individual twisting in ref. ^22^), and introduce this symmetry breaking. Heuristically, the Chern invariant changes from a non-trivial value inside the lattice of cores to a trivial value in the cladding. This change necessitates the local closing of a topological band gap, leading to modes pinned at the edge. This topological argument demonstrates that the edge modes can be engineered to be of arbitrary shape while remaining robust against disorder without any restrictions on geometry or symmetry. Neither pseudo-magnetic fields nor this topological class of effective time-reversal symmetry breaking have been previously realized in fibre.

To see the unique way in which topology characterizes twisted fibre, we begin with the Helmholtz equation for light propagation in a medium with refractive index *n*(*x*, *y*, *z*):

1 $${\nabla }^{2}{\bf{E}}+{k}^{2}{n}^{2}{\bf{E}}=0,$$

where *k* = 2π/*λ* is the free-space wavevector and ∇ is the three-dimensional gradient. We now decompose the electric field **E** = (*ψ*_*x*_, *ψ*_*y*_, *ψ*_*z*_)e^*i*(*βz*−*ωt*)^ into the rapidly oscillating complex phase e^*i*(*βz*−*ωt*)^ with propagation constant *β*, and the slowly varying envelope *ψ*_*i*_(*x*, *y*, *z*). Twisted fibre has an inherently *z*-dependent index *n*(*x*, *y*, *z*), but the transformation into the co-twisting frame,

2 $$x\to x\cos (\alpha z)-y\sin (\alpha z)$$

3 $$y\to x\sin (\alpha z)+y\cos (\alpha z),$$

creates a *z*-independent profile *n*(*x*, *y*), where *α* is the twist rate in radians per metre. However, this coordinate change introduces several effects inherent to optics in twisted frames. Optical activity mixes the two linear polarizations, resulting in circularly polarized eigenstates *ψ*^±^ with propagation constant splitting ±*τ*. As discussed in Supplementary Section I, the torsion *τ* ≈ *α* is constant for our small values of twist *α* and cross-section radius *R*^33–35^, so that optical activity can be absorbed into the eigenvalue for each polarization state.

For each component *ψ* = *ψ*^±^, we write the propagation equation using the paraxial approximation in the co-twisting frame,

4 $${\mathrm{i}}{\partial }_{z}\psi =-\frac{1}{2\beta }{({\nabla }_{\perp }+{\mathrm{i}}{\bf{A}})}^{2}\psi -\frac{{\alpha }^{2}\beta {r}^{2}}{2}\psi -\Delta n(x,y)k\psi ,$$

where ∇_⊥_ is the two-dimensional gradient and Δ*n*(*x*, *y*) is the change in refractive index relative to the silica glass cladding. This equation is analogous to the time-dependent Schrödinger equation for a charged particle in a constant magnetic field with vector potential **A** = *αβ*(*y*, −*x*) and an external harmonic potential *α*^2^*βr*^2^/2, where $r\equiv \sqrt{{x}^{2}+{y}^{2}}$ is the radial coordinate. Although the periodicity of our system in the lab frame suggests a Floquet approach^22^, once we have transformed to the co-twisting frame, we find that the right-hand side of equation (4) features no *z* dependence. This translational invariance allows us to define static eigenmodes and ensures adiabaticity, in contrast to Floquet systems^22^, in which static eigenmodes do not exist. Moving into the co-rotating frame offers a simpler route to understanding our fibre, bypassing Floquet analysis of quasi-energy levels, which would be unavoidable in the lab frame. In our analysis, dynamics in time correspond to propagation along the *z*-direction, and the eigenstate energy corresponds to the relative propagation constant Δ*β*. The twist then breaks the effective time-reversal symmetry $\widehat{{\mathcal{T}}}$, so travelling backwards along the *z*-direction is equivalent to reversing the sign of *α*: $\widehat{{\mathcal{T}}}(z,\alpha ,i)\equiv (-z,\alpha ,-i)=(z,-\alpha ,i)$ (Fig. 1b). In Supplementary Section I, we use Maxwell’s equations in helicoidal coordinates and the paraxial approximation to derive equation (4) and discuss its symmetry properties.

**Extended Data Fig. 1:**
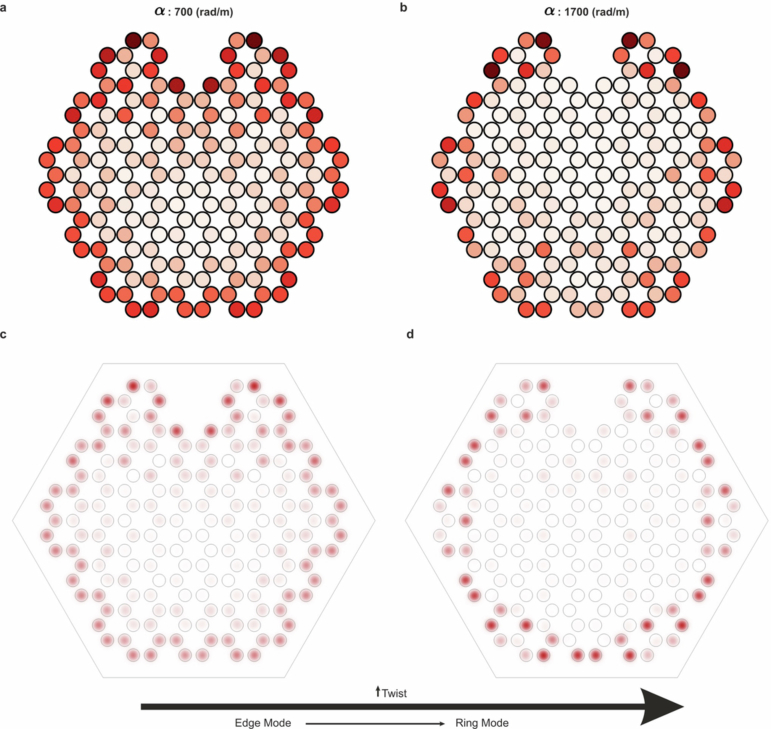
Intensity profiles above and below the topological-to-trivial twist-rate threshold. **a**, Edge mode intensity profile calculated using tight-binding numerics for a fibre with a twist rate of 700 rad m^−1^. The 700 rad m^−1^ twist rate introduces an on-site term that is smaller than (but of the same order as) the coupling strength, so the topological edge-localisation remains. **b**, Intensity profile for a ring-localised mode calculated at a fibre twist rate of 1700 rad m^−1^. A twist rate of 1700 rad m^−1^ introduces an on-site term which is greater than the coupling strength (which defines the topological band gap), so the topological state is destroyed and trivial ring-localised supermodes remain. **c**–**d**, Corresponding data from finite-element simulations: **c**, Intensity profile for an edge mode calculated using finite-element simulation for a fibre with a twist rate of 700 rad m^−1^. **d**, Intensity profile for a ring-localised mode calculated in a finite-element simulation of fibre with a twist rate of 1700 rad m^−1^.

**Extended Data Fig. 2:**
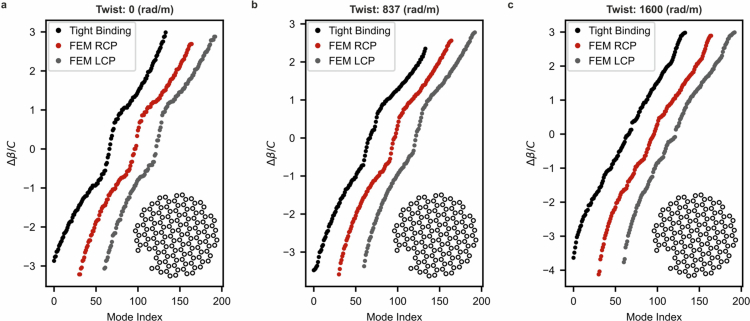
Comparison between tight-binding numerics and finite-element simulation, decomposed into left-circular polarisation LCP and right-circular polarisation RCP. The comparison shows good qualitative agreement between these distinct computational methods across a range of twist rate values. **a**, Change in propagation constants (relative to median) calculated using tight-binding numerics and finite-element simulation with no twist (that is, a twist rate of 0 rad m^−1^) plotted against their mode index to highlight their similarities and differences. The mode indices are found by sorting the propagation constants in ascending order, and we use them here to allow for a clearer comparison between datasets. The finite element method datasets are shifted in mode index to increase visibility and prevent overlap. The change in the LCP propagation constants is calculated relative to the RCP median to highlight the shift due to optical activity induced by twisting the fibre. **b**, Change in propagation constants calculated using tight-binding numerics and finite-element simulation with a twist rate of 837 rad m^−1^, plotted against the mode index. **c**, Change in propagation constants calculated using tight-binding numerics and finite-element simulation with a twist rate of 1600 rad m^−1^, plotted against mode index. All propagation constants are calculated for the fibre cross sections shown in the insets.

**Extended Data Fig. 3:**
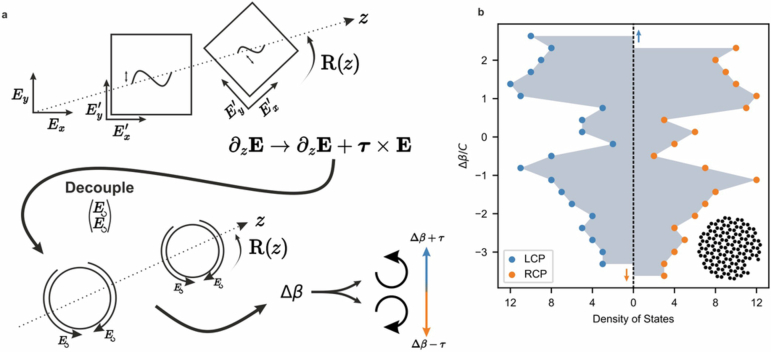
Polarisation effects in twisted optical fibre. **a**, Twisting the fibre leads to a rotation of the electric field along the *z*-axis. Moving into a co-twisting frame reveals a mixing in the *E*_*x*_ and *E*_*y*_ field components. We can return to two uncoupled equations by describing the electric field in the circular polarised basis—where each polarisation is shifted by ± ***τ***. **b**, Finite element simulation is used to calculate supermode data which is projected into left and right circularly polarised bases. Each polarisation has the same general structure in its density of states, with an overall shifting of ± ***τ***.

In the honeycomb lattice of our twisted fibre cross-section, we describe the supported supermodes of *M* coupled cores using coupled mode theory (equivalent to the tight-binding model). Crucially, the vector potential **A** introduces a complex Peierls phase to the (real) coupling strength *C*:

5 $$\Delta {\beta }_{m}{u}_{m}=\mathop{\sum }\limits_{j\ne m}{{\mathrm{e}}}^{{\mathrm{i}}{\bf{A}}\cdot {{\bf{r}}}_{mj}}{C}_{mj}{u}_{\!j}+{D}_{m}{u}_{m},$$

where *u*_*m*_ is the contribution from the *m*th core, **r**_*mj*_ is the position vector between the *m*th and *j*th cores, *D*_*m*_ is the change in propagation constant for a single core owing to the twist, Δ*β*_*m*_ is the change in propagation constant for a supermode and *C*_*mj*_ is the coupling strength between neighbouring cores. This coupled mode theory approach, as described in Supplementary Section I, shows good agreement with our finite-element simulations (Extended Data Figs. 1–3) and serves as an analytical model for understanding light propagation in our fibre.

Equations (4) and (5) show the challenges of characterizing topology in Chern insulators with broken periodicity, which occur not only in our twisted fibre but also in rotating mechanical lattices^36–38^ and cold atomic gases^39–41^. Although the presence of a periodic honeycomb lattice suggests that solutions for modes *ψ* could be found in reciprocal space, the simultaneous presence of the effective vector potential **A** and the scalar potential *α*^2^*βr*^2^/2 destroys translational symmetry. Nevertheless, we experimentally and numerically observe topological edge states and use Kitaev summation^42^ to calculate a local topological marker for our system.

## Results and discussion

**Fig. 2:**
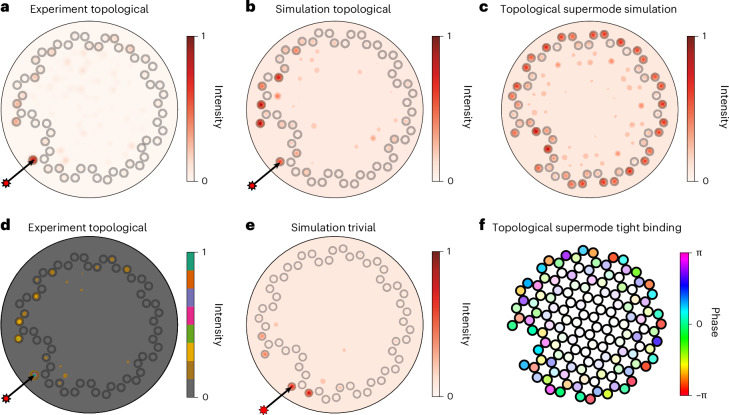
Edge localization of intensity in topological fibre. **a**, Experimental image of intensity output after light is injected into the core (marked by the arrow) and propagated through (24.0 ± 0.5) mm of fibre (data from Fig. 1d without overexposure). **b**, Finite-element simulation of intensity output after light is injected into the marked core and propagated through 23.6 mm of fibre. Although the exact profile is highly dependent on the details of the fabrication, the simulations confirm the experimentally observed edge localization. **c**, Topological eigenmode at the edge of our twisted fibre obtained from a finite-element simulation. **d**, Discretized colourmap of **a**, showing that the intensity distribution is localized at the edge. **e**, Finite-element simulation of intensity output after light is injected into the marked core and propagated through 23.6 mm of untwisted, topologically trivial fibre. Instead of propagating around the edge, light remains localized around the initial injection site. **f**, Topological edge mode calculated from tight-binding numerics, where we plot both the phase (using hue) and the intensity (using saturation). The tight-binding numerics show that the intensity of the edge mode remains localized to the perimeter, and that the phase varies azimuthally, as expected for orbital angular momentum modes.

Experimentally, we probe the topological edge by injecting 1,064 nm light into a single core at the perimeter of our fibre. In Figs. 1d and 2a,d, we show the intensity distribution after coupling light into the marked core and propagating through 24 mm of fibre. Whereas Fig. 2a shows a normalized intensity profile, Fig. 2d uses a discretized colourmap to highlight the intensity in the darker regions. As explained in ‘COMSOL simulations’, our fibre has refractive index ${n}_{{{\rm{SiO}}}_{2}}\approx 1.449$ in the silica glass cladding, and ${n}_{{\rm{doped}}}={n}_{{{\rm{SiO}}}_{2}}+(23.0\pm 0.15)\times 1{0}^{-3}$ in the germanium-doped cores (at experimental wavelengths). We use a geometry with a core-to-core coupling strength of 4,135 m^−1^, twist rate 837 rad m^−1^ and core-to-core distance 3.82 μm. We compare our experimental observations with simulated intensity distributions for our model fibre in the topological (*α* = 837 rad m^−1^) and trivial (*α* = 0 rad m^−1^) cases, calculated for the fabricated fibre parameters using finite-element analysis. Simulations of topological fibre in Fig. 2b reproduce all of the features of the experimental data, in contrast to the topologically trivial model (Fig. 2e), which does not exhibit the same chiral behaviour.

In simulations, we decompose the propagation of light in fibre into supermodes defined by an unchanging transverse profile. In Fig. 2c,f, we plot a supermode that demonstrates the edge localization of propagating light owing to the fibre’s topology. Figure 2c shows an edge mode calculated using finite-element simulation, while Fig. 2f shows the intensity and phase of an edge mode observed in the tight-binding model based on equation (5). These modes arise in the Chern insulator owing to the broken effective time-reversal symmetry and necessarily follow the arbitrary shape along the perimeter. To demonstrate this topological classification, we now directly calculate a real-space Chern marker.

### Real-space Chern marker

**Fig. 3:**
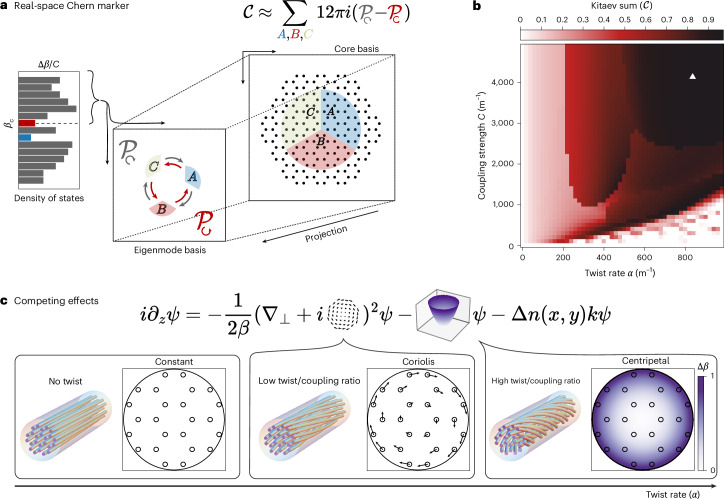
Numerical characterization of topology in fibre. **a**, Schematic explanation of the Kitaev sum used to compute a real-space Chern marker. We define three equal regions (*A*, *B* and *C*) and project the eigenmodes into the states above a selected band gap *β*_*c*_ using operators (${{\mathcal{P}}}_{A},{{\mathcal{P}}}_{B},{{\mathcal{P}}}_{C}$), respectively. The chiral difference ${{\mathcal{P}}}_{\circlearrowright }-{{\mathcal{P}}}_{\circlearrowleft }={{\mathcal{P}}}_{A}{{\mathcal{P}}}_{B}{{\mathcal{P}}}_{C}-{{\mathcal{P}}}_{C}{{\mathcal{P}}}_{B}{{\mathcal{P}}}_{A}$ approximates the Chern number in the bulk of our fibre despite inhomogeneity and finite size (see text and Supplementary Section II for details). **b**, Calculated real-space Chern marker for different values of twist and coupling strength. We plot experimentally relevant parameters to guide topological fibre design and fabrication. We find that the topologically non-trivial region ${\mathcal{C}}=1$ corresponds to both high twist rates (greater than 600 rad m^−1^) and high coupling strength (greater than 3,000 m^−1^), and the triangle in the upper right corresponds to our experimentally fabricated fibre. **c**, Conceptual explanation of the topological transition that occurs as twist rate is increased at a fixed coupling strength, based on equation (4) for guided light in twisted fibre. When the twist is small compared with the coupling strength, the vector-potential (Coriolis) term dominates. Increasing the twist increases the on-site (centripetal) term. At high twist rates, the effect of the centripetal term is to destroy the topology, which we avoid by increasing the coupling strength.

Figure 3a gives a diagrammatic explanation of how the Kitaev sum can be used to calculate a local approximation of a system’s Chern number^42^. In this method, the fibre cores are divided up into three equal regions (*A*, *B* and *C*) in real space. We project the contributions from cores in region *A* (and *B* and *C*) into the eigenstates above a selected cut-off *β*_*c*_ using the operator ${{\mathcal{P}}}_{A}$ (and ${{\mathcal{P}}}_{B},{{\mathcal{P}}}_{C}$, respectively). To find the local Chern current, we use the difference between the two chiral permutations of the product of the three projection operators, ${{\mathcal{P}}}_{\circlearrowright }-{{\mathcal{P}}}_{\circlearrowleft }\equiv {{\mathcal{P}}}_{A}{{\mathcal{P}}}_{B}{{\mathcal{P}}}_{C}-{{\mathcal{P}}}_{C}{{\mathcal{P}}}_{B}{{\mathcal{P}}}_{A}$. To find the local Chern marker, we sum over all cores in the regions *A*, *B* and *C* (see Fig. 3a, details in Supplementary Section II and ref. ^43^).

We compute this local Chern marker for parameter values relevant to our fabricated fibre and plot the resulting phase diagram in Fig. 3b. The two parameters that we experimentally and numerically vary are the inter-core coupling strength (measured in thousands per metre) and the fibre twist rate *α* (measured in hundreds of radians per metre). We find that the local Chern marker ${\mathcal{C}}$ approaches ${\mathcal{C}}=1$ in the region corresponding to both high twist rates (greater than 600 rad m^−1^) and high coupling strengths (greater than 3,000 m^−1^). The white triangle shown in Fig. 3b shows the parameters of the fabricated fibre used in Fig. 2, highlighting its location in the ‘Goldilocks’ zone of the phase diagram. To understand the interplay between these length scales and the topological invariant, we examine the terms in the scalar wave equation (4) induced by the fibre twist.

The twist-dependent terms that arise in the scalar wave equation are drawn schematically in Fig. 3c. The breaking of an effective time-reversal symmetry is entirely due to the vector potential term **A** = *αβ*(*y*, −*x*) (Fig. 3c, middle panel). This term enables a topological band gap owing to the photonic equivalent of the Coriolis force when Maxwell’s equations are re-expressed in the co-rotating frame of the helicoidal fibre. While the band gap can be seen in infinite (or periodic) systems, in finite systems, edge modes appear in this gap. In the finite lattice, the density of states reveals regions with few modes corresponding to the infinite-lattice band gap. For small twists, the low-density-of-states region cannot be observed in our fibre cross-section (or equivalently, the penetration depth of the edge states is larger than fibre cross-section radius *R*). In our experiments, the large values of twist result in a topological region with a size that is on the order of the coupling strength *C*, which also gives the overall bandwidth (setting the fundamental scale for disorder robustness). However, the twist also induces a scalar potential term (Fig. 3c, right panel), which acts to destroy the topological character of the fibre. This scalar potential is the analogue of a centripetal force term in the co-rotating frame of the fibre and breaks periodicity for the honeycomb lattice of cores. To counteract the scalar potential, one could, in principle, pre-compensate for its effect on each core’s propagation constant by scaling core properties (for example, size, shape and material) with position^44^. The magnitude of the scalar potential at the edge of the fibre is given by *V*(*R*) = *α*^2^*βR*^2^/2, and we expect the topological invariant to break down at large twists when *V*(*R*) > *C*. We quantitatively confirm this upper bound on the twist in Supplementary Fig. 9, showing that at high twists, the system instead supports trivial ring-localized modes (Extended Data Fig. 1) that lack topological robustness. The topological fibre requires both the twist and the coupling strength to be large, as in the upper-right corner in the Fig. 3b, which defines the Goldilocks zone where we have focused our fabrication efforts.

**Fig. 4:**
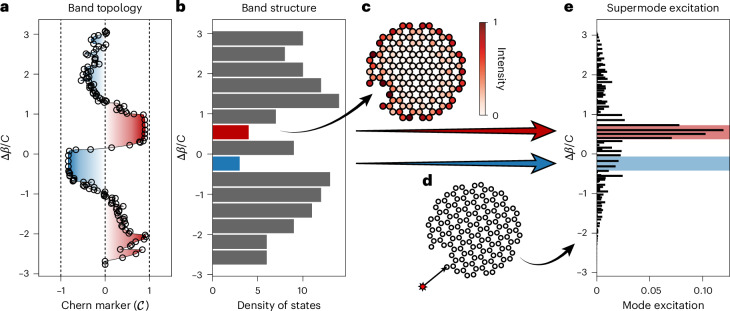
Topology and supported modes. **a**, Local Chern marker as a function of rescaled cut-off propagation constant Δ*β*/*C*. We plot the Chern marker and change in propagation constant of each supported mode to understand the topology of our fibre as a function of the density of states. The two plateaus at ${\mathcal{C}}\approx \pm 1$ correspond to the two gaps surrounding the 0th Landau level of a Dirac material, with equal and opposite Chern numbers. **b**, Histogram showing the density of states as a function of the propagation constant. Topological modes in the less dense (band gap-like) regions are highlighted in red and blue, while modes in the bulk-like regions are shown in grey. The topological edge modes live in the red and blue band gap-analogous regions of the density of states. **c**, Fibre schematic showing a supermode from the upper topological region (${\mathcal{C}}=1$, red), connecting the presence of robust edge modes with the topology of the supported modes. **d**, Diagram showing the core into which light is injected in experiments and finite-element simulations. **e**, Overlap integrals between the supermodes (for each Δ*β*) and the core highlighted in **d**. Injecting into an edge core predominantly excites the topological states, with the mode in the upper band gap region (${\mathcal{C}}=1$, red) excited more than the counter-propagating mode (${\mathcal{C}}=-1$, blue).

We plot the fibre’s supported modes in Fig. 4, resolving both the Chern marker and the density of states as functions of the eigenmodes’ change in propagation constant Δ*β*. In Fig. 4a, we show that the Chern marker is not well defined in the grey sections with high mode density, but shows broad quantized plateaus at ${\mathcal{C}}=\pm 1$ in the lightly populated regions that correspond to band gaps in the infinite case (plotted in red and blue, respectively), as expected from topological band theory. Figure 4b shows that the density of states exhibits two minima for propagation constants corresponding to the topological edge states and the Chern marker plateaus. Although no wavevector-dependent bands exist owing to the non-periodic scalar potential, topological band theory informs how we connect the real-space Chern marker to the edge modes and propagation constants.

### Topological edge mode excitation

**Extended Data Fig. 4:**
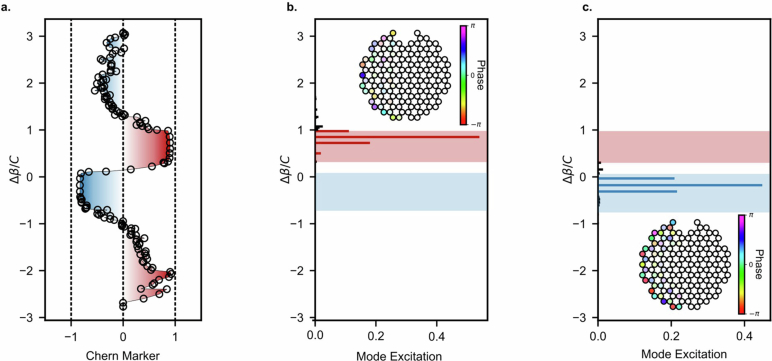
Chiral mode excitation. **a**, Local Chern marker as a function of rescaled cutoff propagation constant *Δβ*/*C*. The two plateaus indicate the regions of the mode structure that contain topological modes with either a positive or negative Chern marker. **b**, Mode overlap between our initial excitation in the ${\mathcal{C}}=1$ regime (shown in the inset) and the rest of the twisted fibre supported modes. In the inset, the initial excitation is plotted. The intensity of the plotted mode is shown by saturation and the hue corresponds to the phase. **c**, Mode overlap between our initial excitation in the ${\mathcal{C}}=-1$ regime (shown in the inset) and the rest of the twisted fibre supported modes. In the inset, the ${\mathcal{C}}=-1$ initial excitation is plotted with saturation corresponding to intensity and hue representing phase.

**Extended Data Fig. 5:**
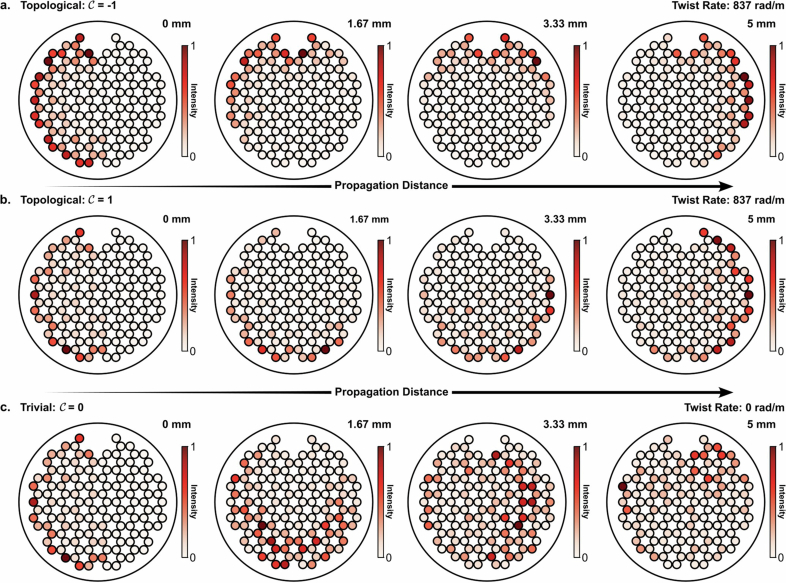
Intensity profile dependence on propagation distance and excited modes. **a**, Intensity profile (shown over 5 mm) when an initial excitation contains exclusively topological ${\mathcal{C}}=-1$ modes. **b**, Intensity profile (shown over 5 mm) when an initial excitation contains exclusively topological ${\mathcal{C}}=1$ modes. **c**, Intensity profile (shown over 5 mm) when an initial excitation contains exclusively trivial ${\mathcal{C}}=0$ modes. The sets of intensity profiles are calculated using our tight-binding numerical model.

**Extended Data Fig. 6:**
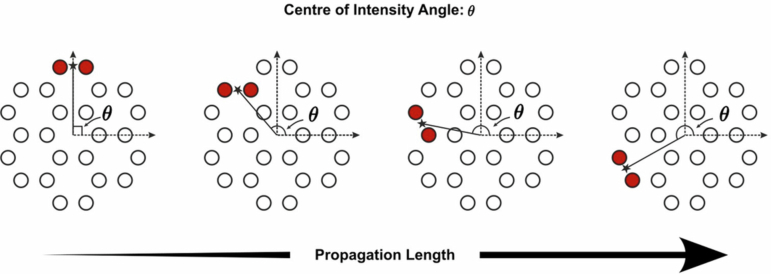
Chiral transport of light intensity. Diagrammatic explanation of how the centre of intensity (⋆) changes as a function of distance. The angle this centre of intensity makes to the origin, *θ*, is used to determine the chiral character of transverse light propagation.

**Extended Data Fig. 7:**
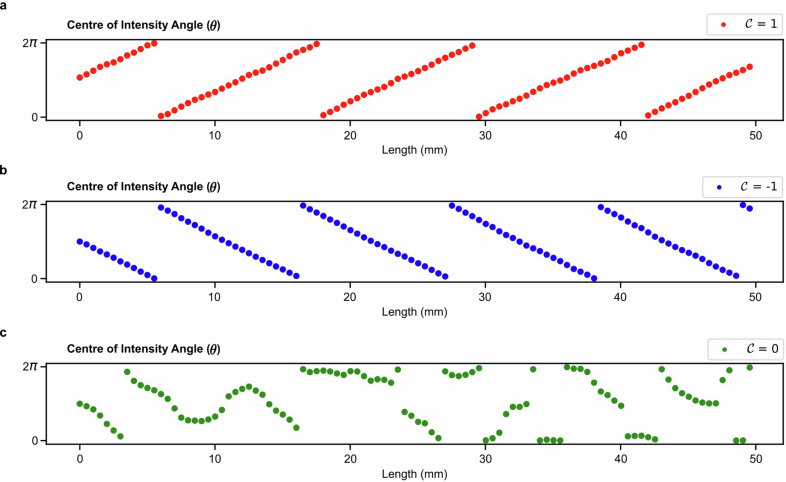
Chiral transport quantified through the change in centre of intensity. The angle that the centre of intensity makes to the origin, *θ*, is plotted as a function of propagation length for three different initial excitations (calculated using our tight-binding numerical model). **a**, An initial excitation created by masking a ${\mathcal{C}}=1$ topological mode (see the inset in Extended Data Fig. 4c for the full field of the initial excitation). **b**, An initial excitation created by masking a ${\mathcal{C}}=-1$ topological mode (see the inset in Extended Data Fig. 4b for the full field of the initial excitation). **c**, An untwisted fibre model is excited using the same excitation as a, to show the expected behaviour of a trivial ${\mathcal{C}}=0$ model. All models are based on our fabricated fibre parameters (a and b feature a twist rate of 837 rad/m, c is untwisted).

**Extended Data Fig. 8:**
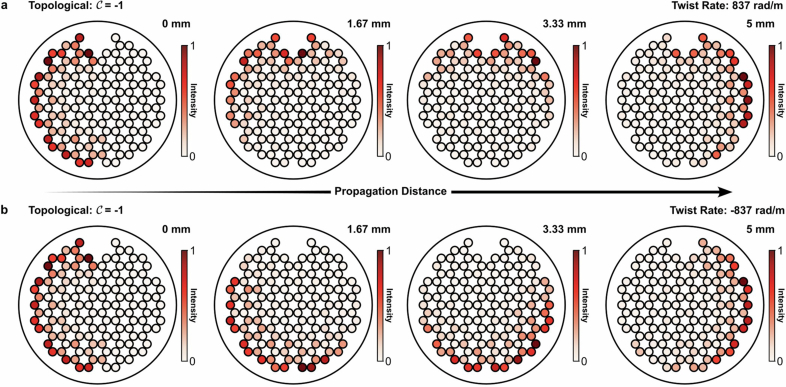
Intensity profile dependence on propagation distance and twist direction. **a**, Clockwise intensity profile (shown over 5 mm) when an initial excitation contains exclusively topological ${\mathcal{C}}=-1$ modes with a positive twist rate (837 rad/m). **b**, Anti-clockwise intensity profile (shown over 5 mm) when an initial excitation contains exclusively topological ${\mathcal{C}}=-1$ modes but the fibre is modeled with a negative twist rate (-837 rad/m). Both sets of intensity profiles are calculated using our tight-binding numerical model.

**Extended Data Fig. 9:**
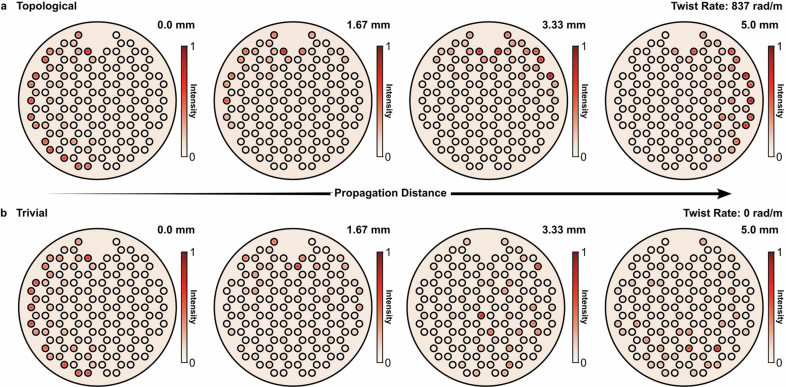
Chiral transport of light intensity in finite-element simulations. The fibre’s supported modes are split into left and right circularly polarised bases and the LCP modes are used to demonstrate chiral propagation. **a**, Half of a topological mode (${\mathcal{C}}=-1$) is used to create an initial excitation that moves clockwise around the fibre cross-section as it propagates. The intensity profile of the propagating state is displayed across 5 mm. **b**, Intensity profile (shown over 5 mm) when the initial excitation in a is incident on an untwisted, topologically trivial, fibre. Both propagation simulations are performed using modes calculated using finite-element methods.

**Extended Data Fig. 10:**
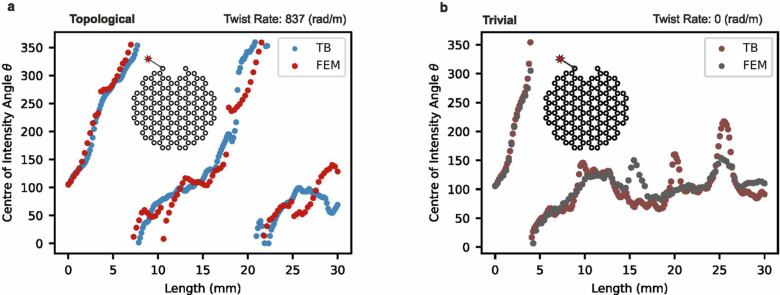
Chiral transport of single-core excitation. **a**, The angle that the centre of intensity makes to the origin, *θ*, is plotted as a function of propagation length for tight-binding (blue) and finite-element (red) models based on our fabricated fibre parameters. Light is initially injected into the core highlighted in the inset. **b**, The angle that the centre of intensity makes to the origin, *θ*, is plotted as a function of propagation length for tight-binding (brown) and finite-element (grey) models based on an untwisted version of our fabricated fibre, where no consistent chiral propagation is observed. Light is initially injected into the core highlighted in the inset.

To understand the experimental results on chiral edge transport, we numerically simulate injecting monochromatic light into an edge core (Fig. 4d) to excite a superposition of supermodes. This superposition can be resolved by computing the overlap integrals for all of the supermodes, plotted in Fig. 4e. Although the supermodes include both bulk and edge states, Fig. 4e shows that most of the light is injected into the chiral edge modes (highlighted regions) that live in the topological gaps. Crucially, the broad range of propagation constants shown in Fig. 4e tells us that both sets of topological states (${\mathcal{C}}=\pm 1$) are excited simultaneously by this single-core injection. These two gaps surround the 0th Landau level and separate it from the ±1 Landau levels. Analogously to graphene^45,46^, this mode structure arises owing to the flattening of the Dirac cone by the twist-induced vector potential. In our setting, each of the chiral edge modes can be distinguished by its orbital angular momentum^47,48^, which can be computed from the number of nodes along the azimuthal direction in, for example, Fig. 2a. Owing to the notch in our fabricated geometry, we excite the counter-propagating chiral edge states with an overall intensity imbalance (exciting more states with a positive Chern marker), which results in a net chiral transport. In Methods, we perform isolated excitations of topological edge modes in the tight-binding model (Extended Data Fig. 4) to observe clean chiral transport in both the upper and lower band gap (Extended Data Figs. 5–7), and with clockwise and anti-clockwise twist rates (Extended Data Fig. 8). In Extended Data Fig. 9, we verify the presence of chiral transport in finite-element simulation, and in Extended Data Fig. 10, we show that chiral transport occurs after a single-core excitation in the tight-binding model.

### Disorder robustness

**Fig. 5:**
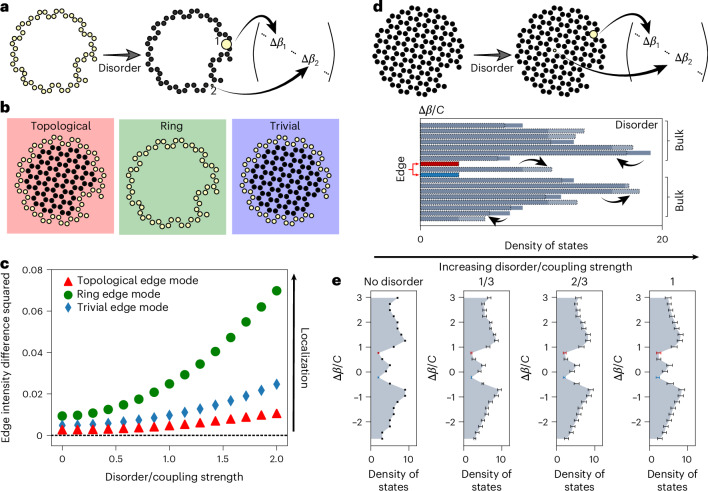
Topological protection against disorder in fibre. **a**, Schematic of disorder in core shape and size, which can be modelled using random terms on the diagonal elements of the coupling matrix, *C*_*jj*_. **b**, Comparison of three hypothetical fibres in the presence of disorder. Our topological twisted fibre (left, red) has twist rate *α* = 837 rad m^−1^ and coupling strength *C* = 4,135 m^−1^, corresponding to the white triangle in Fig. 3b. The ring model (middle, green) is an untwisted array of cores in the same shape as the edge of our topological lattice, which we use to highlight the robustness induced by the topological bulk in our fibre. The trivial model (right, blue) uses a twisted fibre with a large twist, corresponding to a trivial Chern marker (bottom right of diagram Fig. 3b, corresponding to *α* = 1,700 rad m^−1^, *C* = 4,135 m^−1^ and ${\mathcal{C}}\to 0$). **c**, The topological edge mode remains more robust against disorder-induced localization, for all disorder strengths that we consider. We use the edge intensity difference squared to quantify this localization, which we compute by squaring and summing the differences between an edge mode in the disordered system and an idealized edge mode that has equal intensity in every edge core. Each curve corresponds to the most delocalized mode in the absence of disorder, and each point is the average over 1,000 disorder realizations. **d**, Schematic illustration for how a single realization of on-site disorder, introduced across the entire fibre cross-section, changes the density of states for the supported supermodes. **e**, Numerical data for the density of states for increasing disorder strength (left to right). The shaded region shows the density of states in the presence of no disorder, and the error bars correspond to the standard deviation of the density of states across 2,000 realizations of the on-site disorder (see Supplementary Section III for details). Owing to topological robustness, the standard deviation is smaller in the red and blue regions of the density of states, where topological states live (coloured red, blue).

Light transport in chiral edge states is topologically robust. We show two examples of topological robustness that arise in our fibre: Fig. 5a–c shows robustness of chiral edge states against disorder-induced localization, and Fig. 5d,e shows robustness of the propagation constants. In these models, on-site disorder during fibre fabrication changes the size of a core, resulting in a random contribution to the diagonal elements *C*_*jj*_ in the coupling matrix, equation (5) and Fig. 5a.

To show topological robustness in Fig. 5a–c, we compare the topological fibre (left, red panel in Fig. 5b) with two distinct topologically trivial fibres: an untwisted fibre with a ring-like set of cores (middle, green panel in Fig. 5b) and a fibre so over-twisted as to be in a topologically trivial regime (right, blue panel in Fig. 5b). The edge intensity difference squared plotted in Fig. 5c quantifies the modes’ robustness against disorder-induced localization as a function of disorder strength. We calculate the difference in intensity between a chosen mode and an idealized edge mode that has equal intensity in every edge core, and then square and sum these differences to quantify the localizing effect of the disorder. (We provide details in Supplementary Section III, where we also arrive at the same conclusions for bulk disorder and by measuring localization via the inverse participation ratio.) For each of the topological, ring and trivial fibres, we select the most edge-localized mode, introduce disorder and compare how the localization changes. In a trivial state, disorder localizes modes to only a few cores, unlike the topological state, where the chiral supermode must remain delocalized around the entire edge.

Figure 5d,e shows that the propagation constants of topological modes are protected against on-site disorder. The effect of disorder is shown schematically in Fig. 5d: disorder in one core changes the density of states (per unit Δ*β*/*C*) for the supermodes. In Fig. 5e, we use error bars to plot the standard deviation for the density of states across 2,000 different realizations of on-site disorder, with the disorder strength varying from 0 to *C* (see Supplementary Section III for details). We observe that for small disorder strength, the standard deviation in the density of states of the topological edge modes (red and blue) is markedly smaller than the deviations present in the bulk modes (black). We also show that for edge modes, the propagation constant remains topologically protected for a broad range of disorder magnitudes, up to the size of the density of states’ topological region (which is analogous to the width of the band gap), which is set by the nearest-neighbour coupling strength *C*. As expected, once the disorder scale reaches the effective band gap size, topological protection is lost and the standard deviation of the density of edge states becomes comparable to the bulk modes. While the topological robustness of chiral edge modes protects their spatial profiles and propagation constants from disorder of any symmetry class, light propagating in an excited edge state experiences material losses comparable to those in conventional fibre (Supplementary Figs. 14 and 15).

In conclusion, we have fabricated a scalable photonic Chern-insulator fibre that supports the propagation of robust, edge-localized supermodes. We have explored the interaction between the twist and the supported propagation constants to find a Goldilocks zone where the topological character persists even in the presence of a twist-induced parabolic index profile. We compare our robust states with their trivial counterparts, showing how topology protects light in fibre against fabrication disorder. This robustness underlies the promise of topological fibre design to improve signal reliability, protect delicate quantum signals and realize scalable topological fibre lasers by doping with gain media.

## Methods

In this section, we compare the results from our tight-binding model with finite-element-method (FEM) COMSOL simulations and explore light propagation in our tight-binding model and COMSOL simulations.

### COMSOL simulations

To solve for the electromagnetic modes of our twisted fibre cross-section, we combine the cross-sectional geometry of the fibre with the co-twisting coordinate frame. We then introduce a reverse coordinate transform to convert the numerical solutions into the lab frame. The reverse coordinate transform that we use is

6 $$\begin{array}{rcl}x & = & {x}^{{\prime} }\cos (\alpha {z}^{{\prime} })+{y}^{{\prime} }\sin (\alpha {z}^{{\prime} }),\\ y & = & -{x}^{{\prime} }\sin (\alpha {z}^{{\prime} })+{y}^{{\prime} }\cos (\alpha {z}^{{\prime} }),\\ z & = & {z}^{{\prime} },\end{array}$$

where (*x*, *y*, *z*) are the coordinates in the lab frame, (${x}^{{\prime} },{y}^{{\prime} },{z}^{{\prime} }$) are the coordinates in the co-twisting frame and *α* is the twist rate in radians per metre. As highlighted in ref. ^49^, changing the coordinate frame in which the Maxwell equations are described is equivalent to changing the permittivity and permeability tensors that feature in Maxwell’s equations. Instead of solving Maxwell’s equations in a helicoidal frame with scalar permeabilities and permittivities, we introduce the effects of twist into the permittivity and permeability tensors and solve for the supermodes in an untwisted geometry. In the lab frame, the material permittivity is described by *ε* = *ε*_0_*n*², where *n* ≅ 1.449 in the silica glass cladding and *n* ≅ 1.449 + (23±0.15)×10^−3^ in the Germanium-doped cores. Following the approach in refs. ^34,49^, we replace the permittivity (*ε*) and permeability (*μ*) tensors for the untwisted case, with ${\varepsilon }^{{\prime} }=\varepsilon {T}^{-1}$, ${\mu }^{{\prime} }=\mu {T}^{-1}$, where *T* is determined by the Jacobian of the coordinate transform:

7 $$T=\frac{{J}^{T}J}{\det (J)}=\left[\begin{array}{rcl}1 & 0 & \alpha {y}^{{\prime} }\\ 0 & 1 & -\alpha {x}^{{\prime} }\\ \alpha {y}^{{\prime} } & -\alpha {x}^{{\prime} } & 1+{\alpha }^{2}({x}^{{\prime} 2}+{y}^{{\prime} 2})\end{array}\right].$$

After replacing the permittivity and permeability tensors, we can solve for the supermodes of the fibre cross-section. Two of these supermodes, at different levels of twist, are shown in Extended Data Fig. 1c,d. Extended Data Fig. 1c shows a topological edge mode for a 9-ring honeycomb lattice with a single hexagon missing. The FEM solution is in good agreement with our tight-binding numerics, and shows a mode that remains localized to the perimeter, even in the presence of a missing hexagon. As explained in the main text and the ‘Tight-binding numerical solution’ section of Supplementary Information, when we increase the twist rate beyond the topologically protected threshold (that is, when the on-site scalar potential term introduced by the twist is greater than the band gap size), the edge mode is destroyed and only ring-localized modes remain. Extended Data Fig. 1b,d shows a ring-localized mode arising from our solutions. Extended Data Fig. 1b shows the tight-binding prediction in good agreement with the finite-element simulation (Extended Data Fig. 1d). In contrast to the topological model shown in Extended Data Fig. 1a,c, the intensity in the ring-localized mode is not uniform over the whole perimeter of the fibre. The cut-out section at the top of the lattice does not maintain a uniform intensity in the ring-localized cases (Extended Data Fig. 1b,d), but in the topological cases (Extended Data Fig. 1a,c), intensity is localized evenly across all cores at the edge of the fibre.

We can probe the mode structure of our FEM system by solving for the supported propagation constants of our twisted fibre cross-section (see Extended Data Fig. 2 and, for comparison, experimental results in Supplementary Fig. 13). Extended Data Fig. 2a–c shows the comparison between supported propagation constants calculated using finite-element and tight-binding numerics. When there is no twist, both the FEM and tight-binding model show only a single visible band gap-analogous region (Extended Data Fig. 2a). Below the twist disorder threshold, both the tight-binding and COMSOL solutions show two effective band gaps, punctuated with a small group of modes at zero change in propagation constant (Extended Data Fig. 2b). Once the twist-induced disorder is greater than the effective band gap size, the topological gap and associated mode structure are lost in both FEM and tight-binding solutions (Extended Data Fig. 2c).

When solving for our fibre’s supported propagation constants and modes using FEM, we find twice as many solutions as in the tight-binding model (Fig. 2). This occurs because the tight-binding model has assumed one particular polarization. In the FEM solutions, we get modes and propagation constants for both natural polarization bases. As we explain in Supplementary Information (and show in Extended Data Fig. 3), twisting the fibre mixes the *E*_*x*_ and *E*_*y*_ polarization bases, leaving left circularly polarized and right circularly polarized as the more useful separable basis. After splitting the calculated propagation constants into this polarization basis, we see an overall polarization splitting (in propagation constant) of ±*τ*, where *τ* is the torsion introduced by the twist. We derive this result for the optical activity in Supplementary Information.

### Numerical propagation

To simulate propagation in the tight-binding numerics, we take an initial field and by using overlap integrals express this excitation as a sum of supermodes. These supermodes propagate unchanged in *x*- and *y*-coordinates, with the phase evolving as ${e}^{i{\beta }_{s}z}$, where *β*_*s*_ is the propagation constant of each supermode. At each step of propagation, we map the intensity into the original coordinate basis and calculate the resultant intensity profile. A similar process is used for the COMSOL solutions, with the difference that both the supermodes and the initial excitation are now expressed on a 100 × 100 grid that spans the fibre cross-section. By discretizing the supermodes into this 100 × 100 basis, we gain more information about the spatial structure of our solutions while accurately scaling to arbitrary propagation distances.

Investigating propagation dynamics numerically allows us to observe differences between the supported topological and trivial modes on the smallest length scales. As we explain in the main text, it is challenging to excite a single topological mode by simply coupling light into a single core. Instead, if one wishes to see chiral propagation, we must use an initial field with the correct intensity and phase profile. To create this initial field, we take the topological edge mode that we are trying to investigate and we zero the field on half of the fibre cores, and then renormalize it. We show examples of two such initial fields for our twisted fibre model with a twist rate of 837 rad m^−1^ in Extended Data Fig. 4. In Extended Data Fig. 4a,b, we show an initial field that excites modes with a real-space Chern marker ${\mathcal{C}}=1$(${\mathcal{C}}=-1$).

In Extended Data Fig. 5, we show snapshots of intensity as a function of propagation length for the topological and trivial initial excitations. The full dynamics can be seen in Supplementary Video 1. As expected from the tight-binding theory, the topological modes show distinct chiral propagation with a handedness that is governed by the Chern marker of the excited state. Extended Data Fig. 5a shows an initial excitation of a ${\mathcal{C}}=-1$ mode that moves clockwise around the perimeter of the fibre as it propagates, while the ${\mathcal{C}}=1$ mode in Extended Data Fig. 5b moves anti-clockwise. Extended Data Fig. 5c confirms that when the fibre is untwisted, the supported modes are trivial, and light propagation has no chiral character. The characteristic length scales for chiral transport are controlled by the coupling strength between two neighbouring fibre cores: the coupling strength must be large to prevent the topologically deleterious effects of the on-site (centripetal) potential.

To quantify chiral propagation over extended distances, we compute the centre of intensity at each step and track its angular displacement *θ* with respect to the fibre axis (Extended Data Fig. 6). In Extended Data Fig. 7, we plot the change in the centre of intensity as a function of propagation length for states corresponding to ${\mathcal{C}}=1$, ${\mathcal{C}}=-1$ and ${\mathcal{C}}=0$. Extended Data Fig. 7 verifies that the chiral movement of light intensity continues over longer distances, and confirms the qualitative observation that each chiral edge mode propagates with a certain handedness. The direction of the chiral propagation is not solely controlled by which topological mode is excited. The handedness of the fibre twist also controls whether the mode moves clockwise or anti-clockwise. We show this in Extended Data Fig. 8 and Supplementary Video 2, where we compare the same ${\mathcal{C}}=-1$ mode for two fibre models twisted in opposite directions. Reversing the twist direction reverses the handedness of chiral propagation for ${\mathcal{C}}=\pm 1$ modes.

Moving from our simple tight-binding model to finite-element simulations reveals the same topological patterns, but with some differences. If a single topological mode is naively used as an initial excitation (as we show in Supplementary Video 3), the propagation is chiral but contains much more leakage into the bulk. To understand why this happens, we must look at the polarization splitting that occurs in twisted fibre. As we detail in Supplementary Information, when a fibre is twisted, the modes become naturally split between left and right circularly polarized. If we use an initial field that excites both of these polarizations, then while we excite the topological mode of one polarization, we also excite trivial modes of the other. This extra excitation of trivial modes causes light to couple into the bulk and breaks the clean chiral propagation that can be seen in the tight-binding model. However, despite the presence of these bulk excitations, if there is overlap with one of the topological modes, we still see edge-localized intensity (as we highlight in Supplementary Video 3). We can enhance the chirality of the intensity propagation by splitting the COMSOL modes into left and right circularly polarized and then only exciting a topological mode of one polarization. We show this in Extended Data Fig. 9 and Supplementary Video 4, where we propagate a left circularly polarized edge mode excitation and observe clear chiral propagation.

Despite the chiral propagation demonstrated in the numerics, in our experiments, we excite a single edge core with no control over the phase. As shown in Fig. 3 of the main text, injecting light into a single core naturally excites a range of supermodes. When the range of excited supermodes is asymmetrically distributed over the ${\mathcal{C}}=\pm 1$ topological regions, the resultant intensity profile propagates around the edge of the system with a defined chirality. In a system like graphene in a magnetic field, one can set the Fermi level such that only a single chiral edge state can be accessed^46^, but optical systems lack an analogous tuning parameter. Instead, we necessarily excite superpositions of topological modes from both Chern regions (${\mathcal{C}}=\pm 1$). If we excite an equal superposition of these Chern states, the overall propagation of the intensity profile does not have a chiral character. However, if there is greater overlap with modes of one chirality, we observe chiral propagation.

In Extended Data Fig. 10b, we compare the predicted change in *θ* (the angle made to the centre of intensity from the lattice origin) as a function of distance for tight-binding and finite-element propagation. Both the tight-binding and finite-element solutions describe a change in the centre of intensity with a single average direction (increasing *θ*, which corresponds to an anti-clockwise movement of light). This indicates that the unequal excitation of topological edge modes gives rise to chiral transport of light intensity within the fibre cross-section. In Extended Data Fig. 10c, we compare this movement of the centre of light intensity with the numerical predictions of an untwisted fibre over the same length to show that the chirality only arises in the presence of our effective magnetic field. In the trivial case, the angle *θ* no longer moves in one consistent direction.

Our experimental observations show edge localization, which when coupled with our extensive analytical and numerical results show that we have realized a two-dimensional topological invariant in our scalable fibre medium. While observing long-range chiral propagation remains challenging owing to limited control over initial multi-core excitations, this could be addressed in future work by using a spatial light modulator to excite many cores simultaneously with full phase control. Achieving precise control over the initial excitation would be a key step to observing chiral transport over laboratory-scale distances.

## Online content

Any methods, additional references, Nature Portfolio reporting summaries, source data, extended data, supplementary information, acknowledgements, peer review information; details of author contributions and competing interests; and statements of data and code availability are available at 10.1038/s41566-026-01848-9.

## Supplementary information

Supplementary Information Supplementary Discussion, Figs. 1–15 and video captions.

Supplementary Video 1 Simulated intensity transport in the fibre cross-section using the tight-binding model. Left: light coupled into the topological fibre in the ${\mathcal{C}}=1$ edge mode propagates anti-clockwise. Middle: coupling into the ${\mathcal{C}}=-1$ mode yields clockwise propagation. Right: in an untwisted, topologically trivial fibre, the same initial excitation as the ${\mathcal{C}}=1$ case does not show chiral motion.

Supplementary Video 2 Chiral intensity transport and twist direction. Left: in an anti-clockwise-twisted fibre, a ${\mathcal{C}}=-1$ mode propagates clockwise. Middle: in a clockwise-twisted fibre, the same mode propagates anti-clockwise. Right: in an untwisted, trivial fibre, theexcitation does not exhibit chiral motion.

Supplementary Video 3 Finite-element simulation of light propagation in our fibre model. Left: a selection of an E_*x*_ polarized ${\mathcal{C}}=-1$ mode is used as aninitial excitation in a finite-element simulation. This initial profile excites a fibre with a twist rate of 837 rad m^−1^. The intensity moves clockwise around the fibre cross-section, while it propagates along the length of the fibre. Right: the same initial excitation propagates in an untwisted trivial fibre. There is no chiral transport of light intensity in the cross-section.

Supplementary Video 4 Circularly polarized light propagation in our fibre model (finite-element simulation). Left: half of a left-circularly polarized ${\mathcal{C}}=-1$ mode is used as an initial excitation in a finite-element simulation. This initial profile excites a fibre with a twist rate of 837 rad m^−1^. The intensity moves clockwise around the fibre cross-section, while it propagates along the length of the fibre. Right: half of a left-circularly polarized ${\mathcal{C}}=+1$ mode is used as an initial excitation. This initial profile excites a fibre with a twist rate of 837 rad m^−1^. The intensity moves anti-clockwise around the fibre cross-section, while it propagates along the length of the fibre.

Supplementary Video 5 Chiral transport for different twist rates (tight-binding model). Left: a fibre twisted at 200 rad m^−1^ is excited using a mode with areal-space Chern marker of ${\mathcal{C}}=-0.47$. Clockwise edge transport is present but weak due to considerable bulk overlap. Middle: at 400 rad m^−1^, excitation using a ${\mathcal{C}}=-0.74$ shows clearer clockwise chiral transport, with reduced bulkoverlap. Right: at 600 rad m^−1^, excitation using a ${\mathcal{C}}=-0.8$ mode shows strong clockwise chiral transport and minimal bulk overlap.

## Data Availability

The data underlying the results presented in this paper are available via Zenodo at 10.5281/zenodo.17162592 (ref. ^50^).
