# Supplementary material for: Twisted optical fibres as photonic topological insulators
Source: Nat Photonics. 2026 Feb 20;20(3):324–31. doi: 10.1038/s41566-026-01848-9 (PMC12965884; doi:10.1038/s41566-026-01848-9)
Supplement: Supplementary file 1 — Supplementary Discussion, Figs. 1–15 and video captions. [file 41566_2026_1848_MOESM1_ESM.pdf]

nature photonics

Supplementary information https://doi.org/10.1038/s41566-026-01848-9

Twisted optical fibres as photonic topological insulators

In the format provided by the authors and unedited

In this document, we present detailed derivations of the vector and scalar wave equations that describe light propagation in twisted fibre. In addition, we show that twisting the fibre breaks propagation symmetry for an optical mode in a way closely analogous to how an external magnetic field breaks time-reversal symmetry for an electron. Finally, we explore the topological nature of our tight-binding model in greater depth. We show how the measured real-space Chern marker changes as a function of both the vector and scalar potentials, and describe in detail how we can assess the disorder robustness of our supported topological states.

I. FIBRE TWIST AS A VECTOR POTENTIAL

Here we adapt the derivation of the paraxial approximation from the Ref. [1], with the significant difference that in our fibre, the cross-section rotates as a rigid body along the propagation direction. To go to the co-twisting frame, our coordinate transform describes all of the fibre cores twisting helically around a single origin. By contrast, Ref. [1] considers the individual twist of each core—a geometry that is inaccessible in optical fibre, even with state-of-the-art fabrication methods. At the end of the derivation, we arrive at a Schrödinger-like equation [Eq. (4) of the main text] in the presence of a vector potential, where the vector potential is in the symmetric gauge.

We use a combination of analytical and numerical approaches to solve this Schrödinger-like equation in an external gauge field. First, we consider a tight-binding model and convert the vector potential using a Peierls substitution, in order to numerically solve for the supermodes. Our second approach is to use finite-element COMSOL simulations to calculate the supermodes of the fibre cross-section.

A. Assumptions

To begin the derivation, we make the following assumptions, which apply to light propagation in fibre:

- The relative permeability μ_r at optical frequencies is given by $\mu_r = 1$.
- The divergence of the electric field $\mathbf{E}$ vanishes, i.e., $\nabla \cdot \mathbf{E} = -\frac{1}{n^2}\mathbf{E} \cdot \nabla n^2 = -\nabla(\mathbf{E} \cdot \nabla \ln n^2) = 0$. This is because the field is small wherever the change in the refractive index, $\nabla \ln n^2$, is non-zero.
- The envelope of the electric field varies much slower than a period of oscillations [this is called the slowly-varying envelope approximation (SVEA)]: $|\partial_z^2 \mathbf{\Psi}| \ll \beta |\partial_z \mathbf{\Psi}|$.
- The field solution, $\mathbf{E}(x, y, z, t)$ separates into, e.g., $\psi(x, y, z)e^{i(\beta z - \omega t)}\hat{\mathbf{x}}$.
- The change in refractive index is small (known as the weak guidance approximation), so we can expand the square: $n^2 - n_0^2 = (n + n_0)(n - n_0) \approx 2n_0(\Delta n)$.
- Due to the small change in index, the propagation constant can be approximated using $\beta \approx kn_0$, where k is the free-space wavenumber.

B. Vector Wave Equation

Light propagating in a waveguide of arbitary index $n = n(x, y, z)$ is described by the Maxwell equations:

$$\nabla \cdot (n^2\mathbf{E}) = 0, \qquad \nabla \times \mathbf{H} = \epsilon_0 n^2 \partial_t \mathbf{E}, \tag{S1}$$
$$\nabla \cdot \mathbf{H} = 0, \qquad \nabla \times \mathbf{E} = -\mu_0 \partial_t \mathbf{H}. \tag{S2}$$

The vector wave equation is obtained using the common curl-curl identity and assuming $\nabla \cdot \mathbf{E} = 0$:

$$\nabla^2 \mathbf{E} = \mu_0 \varepsilon_0 n^2 \partial_t^2 \mathbf{E}. \tag{S3}$$

We then use the plane-wave solutions, $\mathbf{E}(x, y, z, t) = \mathbf{\Psi}(x, y, z)e^{i(\beta z - \omega t)}$, to write the vector Helmholtz equation:

$$\nabla^2[\mathbf{\Psi}e^{i(\beta z - \omega t)}] = \mu_0 \varepsilon_0 n^2 \partial_t^2 [\mathbf{\Psi}e^{i(\beta z - \omega t)}] \tag{S4}$$
$$= -\omega^2 \mu_0 \varepsilon_0 n^2 [\mathbf{\Psi}e^{i(\beta z - \omega t)}], \tag{S5}$$
$$\implies (\nabla^2 + k^2 n^2)[\mathbf{\Psi}e^{i(\beta z - \omega t)}] = 0. \tag{S6}$$

with Eq. (S6) making use of $\mu_0\varepsilon_0 = \frac{1}{c^2}$ and $k = \frac{\omega}{c}$.

We now make the coordinate transformation into the helicoidal frame, in which the three-dimensional refractive index profile $n(x, y, z)$ of the twisted fibre is transformed into an effective two-dimensional refractive index profile $n(x, y)$. In order to transform into the helicoidal frame, we rotate the electric field into the co-rotating frame via

$$\mathbf{E}(\mathbf{v}) \mapsto R(\tau z) \cdot \mathbf{E}(R^T(\alpha z) \cdot \mathbf{v}), \tag{S7}$$

where $\mathbf{v} = \{x, y, z\}$, and R is the rotation matrix of the helicoidal transformation. We show this rotation schematically in Extended Data Fig. 3**a**. In this notation, $R(\tau z)$ and $R(\alpha z)$ are the helicoidal rotation matrices with arguments τz and αz, respectively, where $\tau = \frac{\alpha}{1+\alpha^2 r^2}$ is the torsion of the helical path and α is the twist rate of the fibre. The two rotation matrices correspond to different rotation angles because the coordinates rotate with the twist rate of the fibre, i.e., by α, whereas, the electric field rotates by the torsion τ [2]. Significantly, for our fibre, the ratio α/τ is close to 1. Explicitly, these transformations take the form:

$$\partial_z \mathbf{E} \mapsto \partial_z \mathbf{E} + \boldsymbol{\tau} \times \mathbf{E} \tag{S8}$$

with

$$\begin{pmatrix} x \\ y \\ z \end{pmatrix} = \underbrace{\begin{pmatrix} c' & s' & 0 \\ -s' & c' & 0 \\ 0 & 0 & 1 \end{pmatrix}}_{R(\alpha z')} \begin{pmatrix} x' \\ y' \\ z' \end{pmatrix}, \qquad \begin{pmatrix} \partial_x \\ \partial_y \\ \partial_z \end{pmatrix} = \underbrace{\begin{pmatrix} c' & s' & 0 \\ -s' & c' & 0 \\ -\alpha y' & \alpha x' & 1 \end{pmatrix}}_{J^{-T}(\alpha z')} \begin{pmatrix} \partial_{x'} \\ \partial_{y'} \\ \partial_{z'} \end{pmatrix}, \tag{S9}$$

where $\boldsymbol{\tau} = \tau\hat{z}$ is the angular rotation rate of the electric field in $\mathrm{rad\,m^{-1}}$, and $c' = \cos \alpha z'$ (similarly, $s' = \sin \alpha z'$). The inverse transpose Jacobian, J^{-T}, is derived from the partial derivatives of R, and describes the coordinate transformation of the derivatives. Substituting the electric field from Eq. (S8) into Eq. (S6) introduces additional terms into the effective Hamiltonian that mix the transverse components of the field,

$$(\nabla^2 + k^2 n^2)[\boldsymbol{\Psi} e^{i(\beta z - \omega t)}] + 2i\beta \boldsymbol{\tau} \times [\boldsymbol{\Psi} e^{i(\beta z - \omega t)}] = 0. \tag{S10}$$

Eq. (S10) contains a second axial derivative of the field, $\partial^2_{z'}[\boldsymbol{\Psi} e^{i(\beta z - \omega t)}]$, which we expand using the product rule,

$$\partial_z^2[\boldsymbol{\Psi} e^{i(\beta z - \omega t)}] = -\beta^2 \boldsymbol{\Psi} e^{i(\beta z - \omega t)} + e^{i(\beta z - \omega t)} \partial_z^2 \boldsymbol{\Psi} + 2i\beta e^{i(\beta z - \omega t)} \partial_z \boldsymbol{\Psi}. \tag{S11}$$

We neglect the second derivative of the field amplitude, $\partial_z^2 \boldsymbol{\Psi}$, using the slowly-varying envelope approximation, $|\partial_z^2 \boldsymbol{\Psi}| \ll \beta |\partial_z \boldsymbol{\Psi}|$. Substituting the expansion Eq. (S11) into Eq. (S10) yields,

$$\nabla_\perp^2 \boldsymbol{\Psi} + 2i\beta(\partial_z \boldsymbol{\Psi} + \boldsymbol{\tau} \times \boldsymbol{\Psi}) + (k^2 n^2 - \beta^2)\boldsymbol{\Psi} = 0. \tag{S12}$$

Here, each term has been divided by the modulating phasor, $e^{i(\beta z - \omega t)}$. Using $\beta \approx k n_0$, we obtain

$$\nabla_\perp^2 \boldsymbol{\Psi} + 2ikn_0(\partial_z \boldsymbol{\Psi} + \boldsymbol{\tau} \times \boldsymbol{\Psi}) + k^2(n^2 - n_0^2)\boldsymbol{\Psi} = 0. \tag{S13}$$

We then approximate $n^2 - n_0^2 = (n + n_0)(n - n_0) \approx 2n_0 \Delta n(x, y, z)$, to find

$$\nabla_\perp^2 \boldsymbol{\Psi} + 2ikn_0(\partial_z \boldsymbol{\Psi} + \boldsymbol{\tau} \times \boldsymbol{\Psi}) + 2k^2 n_0 (\Delta n)\boldsymbol{\Psi} = 0. \tag{S14}$$

Splitting the terms so that the z-derivative is on the right-hand side, we arrive at the analogue of the Schrödinger wave equation,

$$i\partial_z \boldsymbol{\Psi} = -\frac{1}{2\beta}\nabla_\perp^2 \boldsymbol{\Psi} - k(\Delta n)\boldsymbol{\Psi} - i\boldsymbol{\tau} \times \boldsymbol{\Psi}. \tag{S15}$$

We now perform the coordinate transform by substituting equations Eq. (S9) into Eq. (S15). Noticing that the transverse Laplacian is rotation invariant, i.e. $\nabla_\perp^2 \boldsymbol{\Psi} = \nabla_\perp^{'2} \boldsymbol{\Psi}'$, we have,

$$i\partial_{z'} \boldsymbol{\Psi}' = -\frac{1}{2\beta}\nabla_\perp'^2 \boldsymbol{\Psi}' - \alpha \hat{L}_{z'} \boldsymbol{\Psi}' - k(\Delta n')\boldsymbol{\Psi}' - i\boldsymbol{\tau} \times \boldsymbol{\Psi}', \tag{S16}$$

where $\hat{L}_{z'} = -i(x'\partial_{y'} - y'\partial_{x'})$ is the z' component of the angular momentum operator. These terms arise due to the action of the Jacobian on the z-derivative in Eq. (S9):

$$\partial_z = \partial_{z'} - \alpha y' \partial_{x'} + \alpha x' \partial_{y'}. \tag{S17}$$

We rewrite two of the terms (the Laplacian and the angular momentum terms) from Eq. (S16), by completing the square, to obtain terms of the form of a vector potential and a parabolic scalar potential, i.e.,

$$-\frac{1}{2\beta}\nabla'^2_\perp\mathbf{\Psi'} - \alpha\hat{L}_{z'}\mathbf{\Psi'} = -\frac{1}{2\beta}(\nabla'_\perp + i\mathbf{A})^2\mathbf{\Psi'} - \frac{\alpha^2\beta r'^2}{2}\mathbf{\Psi'}, \tag{S18}$$

where we define $\mathbf{A} \equiv \alpha\beta(y', -x')$ and $r'^2 = x'^2 + y'^2$. Substituting Eq. (S18) into Eq. (S16), we obtain

$$\begin{aligned} i\partial_{z'}\mathbf{\Psi'} &= -\frac{1}{2\beta}(\nabla' + i\mathbf{A})^2\mathbf{\Psi'} - \frac{\alpha^2\beta r'^2}{2}\mathbf{\Psi'} - k\Delta n'\mathbf{\Psi'} - i\boldsymbol{\tau}\times\mathbf{\Psi'} \\ \mathbf{A} &= \alpha\beta(y', -x') \end{aligned} \tag{S19}$$

To treat the polarisation degrees of freedom in this vector equation, we go into the circular polarisation basis [3],

$$\mathbf{\Psi}^\pm = \frac{\Psi_x \mp i\Psi_y}{\sqrt{2}}, \qquad |\pm\rangle = \frac{\hat{x} \pm i\hat{y}}{\sqrt{2}}, \tag{S20}$$

which allows us to re-express Eq. (S19),

$$i\partial_{z'}\Psi^\pm = -\frac{1}{2\beta}(\nabla'_\perp + i\mathbf{A})^2\Psi^\pm - \frac{\alpha^2\beta r'^2}{2}\Psi^\pm - k(\Delta n')\Psi^\pm \mp \tau\Psi^\pm \tag{S21}$$

$$= -\frac{1}{2\beta}(\nabla'_\perp + i\mathbf{A})^2\Psi^\pm - \frac{\alpha^2\beta r'^2}{2}\Psi^\pm - (\Delta\beta \pm \tau)\Psi^\pm \tag{S22}$$

where the equation for the right-circularly polarised eigenvector Ψ^+ corresponds to the top sign, and the equation for Ψ^- corresponds to the bottom sign. In our fibre, the twist rate α is much greater than the radial position of each core r, so the spatial variation of $\tau(=\frac{\alpha}{1+\alpha^2r^2})$ can be neglected, and Eq. (S22) reveals the presence of circular birefringence. Each of the decoupled equations for each component of the polarisation in this basis is subject to the same vector-potential and scalar-potential terms. The effect of the twist on the polarisation can be described by a relative constant shift of the respective eigenvalues (see Extended Data Fig. 3**b** for numerical verification of this effect for the specific geometry of our fibre). By considering only one of the polarisation components, we describe the system using a scalar wave equation of the form

$$i\partial_{z'}\psi' = -\frac{1}{2\beta}(\nabla'_\perp + i\mathbf{A})^2\psi' - \frac{\alpha^2\beta r'^2}{2}\psi' - k(\Delta n')\psi', \tag{S23}$$

$$\mathbf{A} = \alpha\beta(y', -x'), \tag{S24}$$

where the circular birefringence terms have been absorbed as a constant in the index $\Delta n'$. Dropping the $'$ symbols for convenience, we arrive at Eq. (4) in the manuscript,

$$i\partial_z\psi = -\frac{1}{2\beta}(\nabla_\perp + i\mathbf{A})^2\psi - \frac{\alpha^2\beta r^2}{2}\psi - \Delta n(x,y)k\psi \tag{S25}$$

C. Time-reversal symmetry breaking

Here, we consider the symmetry properties of the scalar wave equation (S23), and define a natural time-reversal symmetry which is broken due to the twist in our fibre. The coordinate transform from the laboratory frame to a frame that co-rotates with the waveguides is given by Eq. (S9). In our fibre, the propagation direction, z, plays the role of time in the analogous Schrödinger equation. We use this analogy to define a time-reversal operator [4]:

$$\hat{\mathcal{T}}: \quad z \longmapsto -z, \;\; i \mapsto -i. \tag{S26}$$

We note that performing the time-reversal operation $\hat{\mathcal{T}}$ on the helicoidal coordinate system, Eq. (S9), is the same as reversing the twist, $\alpha \mapsto -\alpha$:

$$x'(\alpha) \longmapsto x\cos(\alpha[-z]) - y\sin(\alpha[-z]) = x\cos([-\alpha]z) - y\sin([-\alpha]z) \equiv x'(-\alpha) \tag{S27}$$

$$y'(\alpha) \longmapsto x\sin(\alpha[-z]) + y\cos(\alpha[-z]) = x\sin([-\alpha]z) + y\cos([-\alpha]z) \equiv y'(-\alpha) \tag{S28}$$

$$z' \longmapsto -z'. \tag{S29}$$

Due to the local z'-translation invariance of the Hamiltonian in Eq (S23), the solution ψ' is a function only of the transverse helicoidal coordinates, x' and y'. Time reversal then implies

$$\psi'(x'(\alpha), y'(\alpha)) \longmapsto \psi'(x'(-\alpha), y'(-\alpha)). \tag{S30}$$

When time-reversal is applied to Eq. (S23), z-reversal is equivalent to Eq. (S30), whereas complex conjugation affects the vector-potential term:

$$(\nabla'_\perp + i\mathbf{A}(\alpha))^2 \longmapsto (\nabla'_\perp - i\mathbf{A}(\alpha))^2 \equiv (\nabla'_\perp + i\mathbf{A}(-\alpha))^2. \tag{S31}$$

In combination, these transformations imply that Eq. (S23) obeys time-reversal symmetry only if the twist is also reversed simultaneously,

$$\hat{\mathcal{T}}\{\hat{H}(\alpha)\psi'(\alpha)\} \longmapsto \hat{H}(-\alpha)\psi'(-\alpha). \tag{S32}$$

In other words, without the vector potential, the system would be time-reversal symmetric, but the vector potential $\mathbf{A}$ explicitly breaks this time-reversal symmetry.

D. Multicore Twisted Fibre

Here we derive Eq. (5) in the main text. When considering a fibre cross-section containing multiple cores, we can use coupled mode theory (equivalently, the tight-binding model) to calculate the fibre's supported supermodes [5] using the following equation:

$$\beta\mathbf{u} = \mathrm{C}\mathbf{u}, \tag{S33}$$

where β is the propagation constant of the supermode, $\mathbf{u}$ is a vector containing u_i, which is the amplitude of the transverse field profile in the i-th core. C is the coupling matrix that features the individual core β_0 on the diagonal and the coupling coefficients between cores on the off-diagonal.

In the untwisted case, we can express Eq. (S33) as

$$\Delta\beta\mathbf{u} = \mathrm{C}\mathbf{u}, \tag{S34}$$

where $\Delta\beta$ describes the change in propagation constant of a supermode from the propagation constant of an equivalent uncoupled core. By making the change from β to $\Delta\beta$, the diagonal of the coupling matrix becomes zero.

To introduce the effects of twist into our supermode equation, we add a twist-dependent change in propagation constant for each core (due to the scalar potential) and we introduce complex phases to the coupling coefficients (due to the vector potential). The change in propagation constant is a consequence of geometry – as a fibre is twisted, light on the outside travels further to cover the length of the fibre. This change in path length gives rise to a change in propagation constant that is dependent on each core's radial position [6]. As the change in radial path length is different for each core, we introduce the difference between the twisted and untwisted propagation constant along the diagonal of our coupling matrix.

To reintroduce the vector potential, we use a Peierls phase to modify the coupling of light between cores [7–9]. The Peierls phase is introduced into each coupling coefficient as,

$$C_{mj} \longmapsto e^{i\mathbf{A}\cdot\mathbf{r}_{mj}} C_{mj}, \tag{S35}$$

which mirrors how the effects of a magnetic field are introduced into a tight-binding model of, e.g., graphene [10].

We can now express each of the coupled equations as:

$$\Delta\beta_m u_m = \sum_{j\neq m} e^{i\mathbf{A}\cdot\mathbf{r}_{mj}} C_{mj} u_j + D_m u_m, \tag{S36}$$

where C_{mj} is the untwisted coupling coefficient which can be calculated from the overlap between two untwisted cores and D_m is the change in propagation constant for a given core when it is twisted.

E. Tight-Binding Numerical Solution

To find the modes supported by our fibre, we express the solution in terms of the single-core modes that exist in the cross-section. This coupled mode theory approach (also called the tight-binding model) simplifies the numerics and enables us to find our fibre's supermodes from the eigenvalue problem set up in Eq. (5) in the main text. When only considering nearest neighbours, the nonzero terms in the coupling matrix C for one mode are given by:

$$\begin{aligned}\Delta\beta_m u_m =& C_{m,m+a_1} e^{i\mathbf{A}(\mathbf{m}+\mathbf{a}_1/2)\cdot\mathbf{a}_1} u_{m+a_1} + C_{m,m+a_2} e^{i\mathbf{A}(\mathbf{m}+\mathbf{a}_2/2)\cdot\mathbf{a}_2} u_{m+a_2} + \\ & C_{m,m+a_3} e^{i\mathbf{A}(\mathbf{m}+\mathbf{a}_3/2)\cdot\mathbf{a}_3} u_{m+a_3} + D_m u_m.\end{aligned} \tag{S37}$$

The first three terms on the right-hand side of Eq. (S37) describe the complex couplings between a chosen core (labeled m and located at $\mathbf{m}$) and its nearest neighbours (shown in Fig. S1). The coupling strength, $C_{m,m+1}$, describes the rate (in units of inverse distance), at which light couples from core m to core $m+1$ when the fibre is untwisted. The coupling strength is governed by the spatial overlap between the supported modes in each core, and is fixed for a given wavelength of light. The Peierls phase is found by solving for the vector potential at the mid-point between each pair of cores and computing the dot product with the lattice vector separating these two cores (corresponding to $\mathbf{a}_{1,2,3}$). The final term in Eq. (S37) describes the on-site radially-dependent change in propagation constant that a core experiences due to the twist. This on-site term is approximated as $D_m = \beta_0\sqrt{1+\alpha^2 r_m^2} - \beta_0$, where β_0 is the propagation for an untwisted core of the same size and shape [6].

We can model an idealised version of our fibre by first neglecting the on-site term D_m. Figure S2**a** shows the computed propagation constants for our model fibre under this assumption. Varying the twist rate of the fibre changes both the eigenvalues (propagation constants) and eigenmodes (supermodes of the system). First, the trivial (quasi-)band gap closes and two new effective band gaps appear as Landau levels become better defined. As expected from topological band theory in graphene, these gaps host robust topological edge modes that enable chiral propagation around the edge of the fibre cross-section.

Before we consider these states in greater detail, we first add back the on-site potential term that arises in a multicore fibre due to twist. When the on-site terms are introduced along the diagonal of the coupling matrix, if these terms are smaller than the size of the topological region in the density of states (which approaches the coupling strength as the twist is increased), the on-site terms do not change the topological character of the fibre. However, if the on-site terms are greater than the coupling strength (and effective band gap size), the topology breaks down and the system becomes topologically trivial. The coupling strength therefore sets the scale for the topological robustness, and the twist rate at which the on-site terms become greater than the coupling strength forms a natural threshold for topological behaviour. We show in Fig. S2**b** how the supported propagation constants change above and below this twist rate threshold.

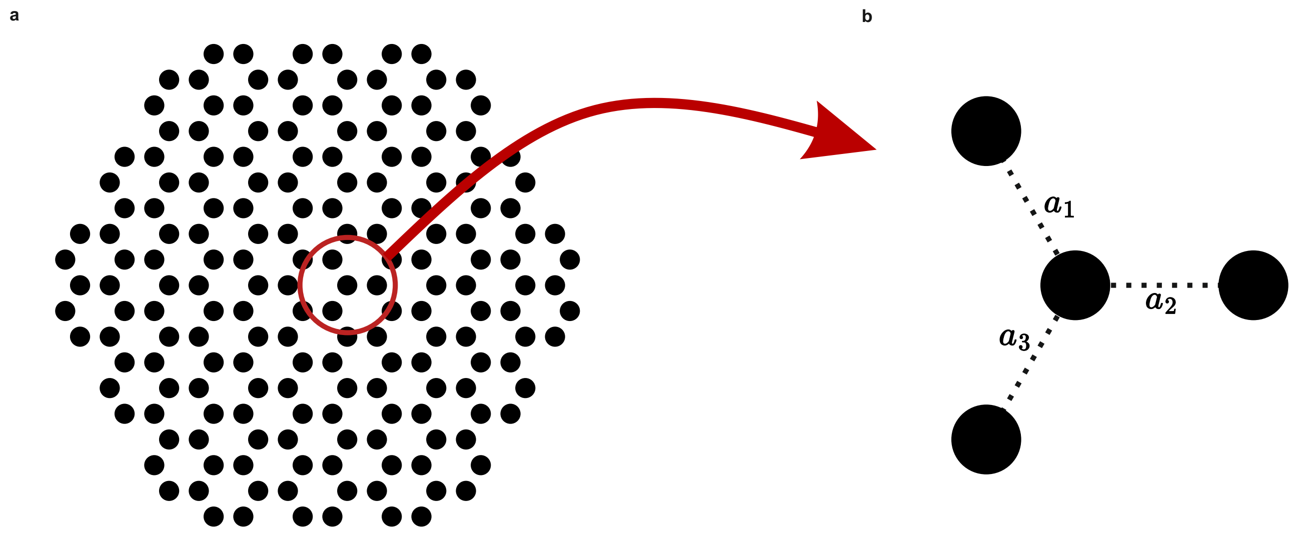

Fig. S1. **a**, Diagram of a 9-ring honeycomb lattice. Each black circle corresponds to a Ge-doped fibre core. **b**, Couplings between nearest neighbours are labelled with their associated lattice vectors from the central core.

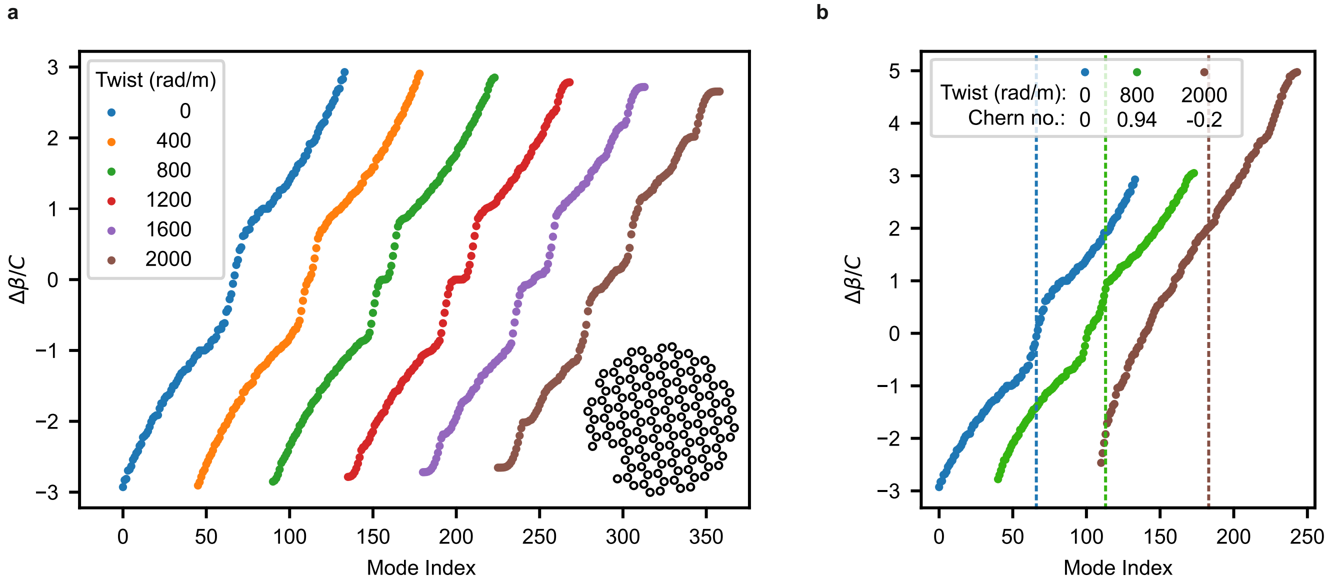

Fig. S2. **a**, Effect of twist rate on the supported propagation constants, when no on-site terms are considered (calculated using tight-binding numerics). The propagation constants are sorted from lowest to highest eigenvalue $\Delta\beta$ and the mode index corresponds to their position in this order. By plotting the supported propagation constants as a function of mode index, we observe how the effective band gaps (which correspond to a large spacing between propagation constants) change as a function of the fibre twist rate. As the twist rate increases, the two supported effective band gaps grow from the original trivial (quasi-)band gap which is present in a Dirac material without twist. Each dataset is shifted horizontally for improved visibility. **b**, Propagation constants calculated using the tight-binding numerics, including on-site potential terms, for three values of the twist rate: zero twist, twist below the calculated threshold for over-twisting, and twist above this threshold. The legend shows the twist rate and the computed real-space Chern marker.

Figure S3 shows the behaviour of the edge modes and mode structures below the twist-rate threshold. Figure S3**a** shows three sets of supported propagation constants for three different values of twist rate (plotted against mode index for visibility). As the twist rate increases, the zeroth Landau level becomes more clearly defined and the gaps above and below can be more easily identified. By looking at modes in the lower gap, we can observe the impact of the twist rate on the intensity profiles of the topological supermodes. In Figure S3**b-d**, we take the same supermode from each dataset and plot the intensity profile. Comparing the intensity profile for each twist rate reveals characteristic changes in the topological edge localisation – increasing the fibre twist rate increases the edge-localisation of the supported topological supermodes. In a finite lattice, topological edge states have an associated width called the penetration depth [10, 11]. As we vary the twist (and consequently the vector potential) the penetration depth of the edge modes gets smaller and they become more localised to the cores at the perimeter of the system. To enable clear observation of the topological edge modes, the penetration depth must be sufficiently small, and thus the twist rate sufficiently high. If the penetration depth is large, the topological mode will have a greater overlap with cores in the bulk of the fibre. This greater overlap means that light coupled into the edge of the fibre will not stay robustly localised, and instead light will couple into the bulk of system and the characteristic topological localisation will be obscured. In our system, we balance our requirement of a small penetration depth with the parabolic scalar potential term that we introduce due to the twist.

Twisting our fibre introduces both on-site (centripetal) and vector potential terms into the scalar wave equation. To create a topologically non-trivial fibre, we must ensure that the topological gap introduced into the density of states by the vector potential is greater than the on-site (centripetal) term. However, as the twist rate increases, the on-site (centripetal) term grows faster than the topological-gap size, leaving the fibre topologically trivial at high twist rates. When twisting a fibre, the supported propagation constants of each core begin to vary as a function of radial distance. As supermodes can only form from cores that have similar propagation constants, once the on-site potential dominates and the fibre becomes topologically trivial, the supermodes form ring shapes at fixed radii. These ring-localised modes are no longer protected by a topological band gap, but are trivially localised into ring shapes. We demonstrate the difference between topological edge states and ring-localised modes in Extended Data Fig. 1**a** and Extended Data Fig. 1**b** and compare our tight-binding predictions to the finite-element solutions found using COMSOL Multiphysics.

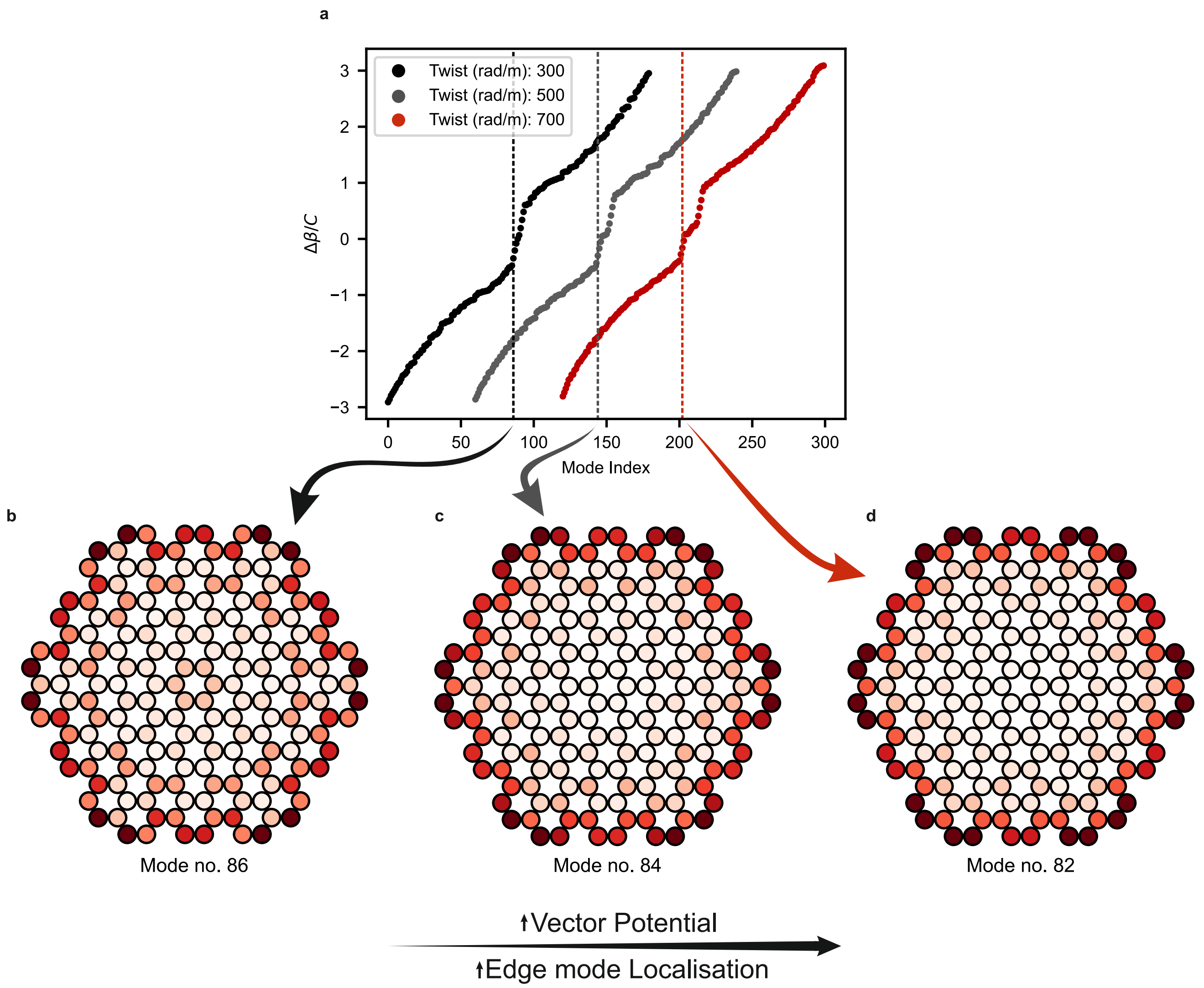

Fig. S3. Supported propagation constants and edge mode penetration depth as a function of twist. **a**, Supported propagation constants for three different fibre twist rates are plotted against their mode index (with a horizontal shift for readability). The mode index labels the supermodes in ascending order (as in Fig. S2). The dotted lines correspond to the same edge mode in each fibre realisation, and their intensity profiles are plotted below. **b**, Intensity profile of the selected mode from the fibre with the lowest twist rate in **a**, for which the edge mode becomes visible. **c**–**d**, Intensity profiles of the selected mode at two alternative twist rates in **a**, for which the edge mode have smaller penetration depths than in **b**.

II. REAL-SPACE CHERN MARKER CALCULATION

In this section, we define the real-space Chern marker, compute the Chern marker as a function of twist amplitude, and show that in our fibre, there are two band gaps characterised by opposite Chern numbers, in analogy with graphene in a magnetic field.

As highlighted in the main text, Chern numbers are typically defined in systems that feature the translational symmetry of a periodic lattice. Due to this symmetry, a repeating unit cell and reciprocal space can be defined. In such systems, the Chern number is the degree of the map between the reciprocal space torus and the unit sphere on which the eigenvectors of the Hamiltonian (or, in the photonic case, the coupling matrix) lives. Although usually defined in periodic systems, it is also well established that systems without translational invariance can possess non-zero topological invariants, such as the Chern numbers, which can be computed using real-space methods [12, 13].

Calculating the Chern number of a system without translational symmetry requires a method that works in real

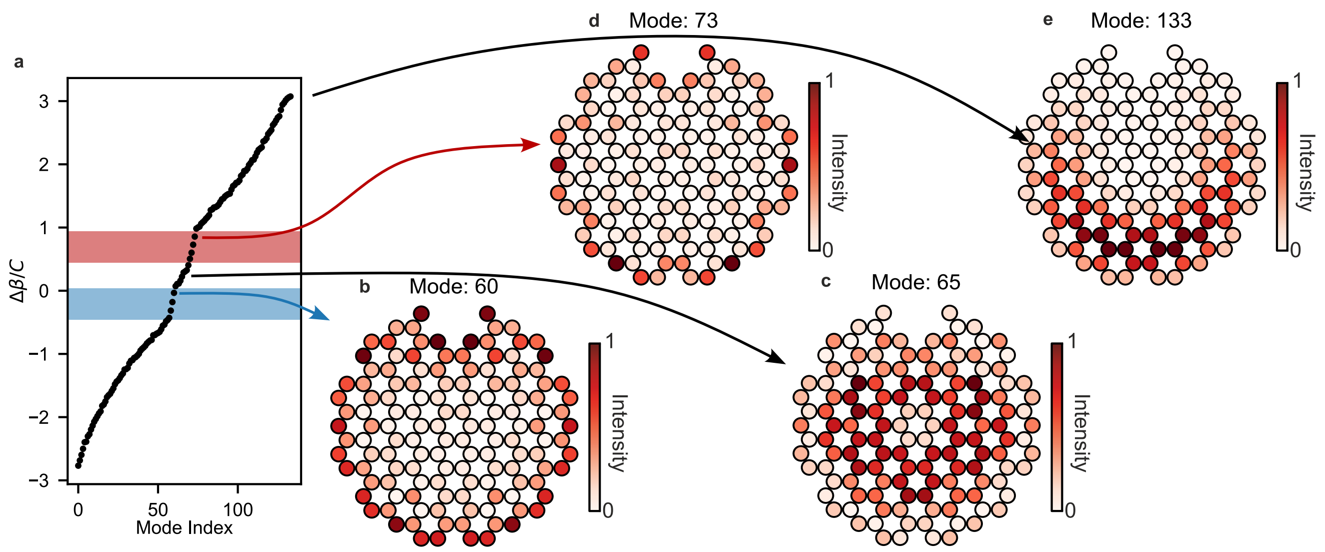

Fig. S4. Intensity profiles of supported modes **a**, The supported propagation constants of our model fibre (with a twist rate of 837 rad/m and the geometry shown in **b**). Two regions of topological modes are highlighted - modes in the red (blue) region have a local Chern marker $\mathcal{C} \approx 1$ ($\mathcal{C} \approx -1$). **b**, Intensity profile for mode 60 (in the blue region). **c**, Intensity profile of mode 65 (in the zeroth Landau level). **d**, Intensity profile of mode 73 (in the red region). **e**, Intensity profile of mode 133 (the greatest change due to the on-site term)

space, such as the Kitaev sum. The Kitaev sum measures a band's integrated chirality within a finite-size system. By defining a projection operator, $\mathcal{P}$, which selects states above a band-gap defined by a cutoff propagation constant, β_c, a non-integer-valued approximation of the local Chern marker $\mathcal{C}$ can be obtained. Following the derivation laid out in Refs. [12, 13], we start by defining the projection operator $\mathcal{P}$:

$$\mathcal{P} = \sum_{\beta > \beta_c} |u_\beta\rangle\langle u_\beta|, \tag{S38}$$

where $|u_\beta\rangle$ is an eigenvector of the fibre's coupling matrix. For each supported propagation constant above the cut off β_c, the outer product between the associated eigenvector and itself forms a projection operator matrix. Summing these matrices creates a matrix that, when multiplied by a vector describing the light intensity in each core, returns solely the contributions from the eigenvectors above the cut off propagation constant β_c.

The elements $\mathcal{P}_{jk}$ of this projection matrix describe the strength with which cores j, k contribute to the same eigenmodes (above the cut off). If the supported eigenvectors had only real-valued amplitudes, the projection matrix would be symmetric and $\mathcal{P}_{jk} = \mathcal{P}_{kj}$, but due to the complex amplitude of the eigenvectors, directional dependent mode overlap can arise. To connect the projections to the underlying topology, we define three regions (A,B,C), pick a core (j, k, l) from each region, calculate the product of the projections in the $j \to k \to l$ direction ($\mathcal{P}_{jk}\mathcal{P}_{kl}\mathcal{P}_{lj}$), and then subtract the product of the projections in the opposite $(j \to l \to k)$ direction ($\mathcal{P}_{jl}\mathcal{P}_{lk}\mathcal{P}_{kj}$). To simplify the final equation we can write these intermediate projection operators as:

$$\mathcal{P}_{\circlearrowright} = \mathcal{P}_{jk}\mathcal{P}_{kl}\mathcal{P}_{lj} \tag{S39}$$

$$\mathcal{P}_{\circlearrowleft} = \mathcal{P}_{jl}\mathcal{P}_{lk}\mathcal{P}_{kj}. \tag{S40}$$

For each set of cores, we find whether the projections of a particular direction contain greater contributions to the modes above a cut off. As we sum over all combinations of cores from each region, we approach a constant value. After multiplying by $12\pi i$, the approached value is an approximation of the Chern number for the system [12, 13]

$$\mathcal{C}(\mathcal{P}) = \sum_{j\in A}\sum_{k\in B}\sum_{l\in C} 12\pi i(\mathcal{P}_{\circlearrowright} - \mathcal{P}_{\circlearrowleft}), \tag{S41}$$

The regions A, B, C are three non-overlapping regions defined on the lattice in real space, and are shown on the lattice inset in Fig. S9**b**.

Using this real-space method, we investigate the topology of our multicore fibre system. By measuring the value of the Chern marker for the upper band (above β_c in Fig. S9**a**), we gain insight into how the topology changes as

a function of twist rate. Fig. S9**b** shows how the calculated Chern marker, computed for the lattice shown in the inset, changes as function of twist. The graph shows that for both small twist rates and large twist rates, the fibre is topologically trivial, but for intermediate values of the twist rate, the Chern marker indicates non-trivial topological character. For this plot, the nearest neighbour coupling (in the untwisted case) is $4135\,\mathrm{m}^{-1}$, which corresponds to the experimentally fabricated fibre at an optical wavelength of $1\,\mu\mathrm{m}$. The bottom horizontal axis is labelled with the non-dimensionalised amplitude of the centripetal term and the top horizontal axis is labelled with the corresponding non-dimensionalised amplitude of the vector potential, both rescaled by the coupling strength. The twist amplitude simultaneously sets the values along both of the horizontal axes. As the twist increases (i.e., as we move to the right along the horizontal axis) both the centripetal and the vector potential terms increase, and the Chern marker becomes non-zero. However, once the centripetal term becomes greater than the coupling strength (dotted line), the topology breaks down and the Chern marker rapidly decreases.

So far, we have only considered a single Chern marker computation for the whole of the band structure, by considering only a single band gap (and a single cutoff) for the projection operators. In some models of Chern insulators such as the Haldane model, this is sufficient because there is only a single band gap, with the bands above and below the gap characterised by equal and opposite Chern numbers. However, our fibre is more analogous to the Landau levels in graphene, and we instead observe not only an upper and a lower band, but also a small middle band of states, which creates two separate band gaps. To calculate the Chern numbers of each band independently, we scan the cutoff β_c across all values of the propagation constant.

Figure S10 shows the Chern numbers for each band in a model fibre system that features a complete lattice (no cut out region) and coupling strength ($6182\,\mathrm{m}^{-1}$). Figure S10**a** shows the calculated Chern number on the horizontal axis, as a function of the cutoff propagation constant β_c (rescaled by nearest neighbor coupling C). As a check, the results in Fig. S10**a** show that when the cutoff is above or below all of the bands, the Chern marker is zero. If the cutoff is in one of the band gaps, the Chern marker obtains a non-zero value. We observe that inside the lower band gap, the Chern marker is computed to be -1, and the Chern marker keeps this near-integer value across the whole plateau corresponding to the band gap. This is because the edge modes that exist within the band gap are localised to the edge of the lattice, at sites that are not included within the (bulk) Kitaev sum. Crossing the small band of states near $\Delta\beta = 0$, we see that the Chern number flips to 1, where it remains across the plateau corresponding to the upper band gap. This plot shows that our twisted fibre is characterised by two band gaps with equal and opposite Chern numbers, analogously to graphene in a magnetic field [10].

III. ROBUSTNESS TO DISORDER

In this section, we present the details that demonstrate the fibre's topological robustness, as described in the main text.

We first explore how disorder changes the localisation of the supported supermodes. To compare the localising effects of fabrication-relevant disorder, we augment the coupling matrix of five comparable fibre models (compare to Fig. 4**b** in the main text, where we include only three of these models): our fabricated topological fibre, an untwisted trivial version of our fabricated fibre, an overly-twisted (1700 rad/m) trivial version of our fabricated fibre, an untwisted fibre that only contains cores at the outer edge, and a twisted (837 rad/m) fibre only featuring edge cores. In order to make a fair comparison, we ensure the edge cores in all coupling matrices are subject to the same disorder. The disorder is introduced by drawing random values from a uniform distribution between $\pm 1/2$, multiplying these values by the disorder strength, and adding this disorder on the diagonal of the coupling matrix in our coupled mode theory (i.e., tight-binding model). Diagonalising the new coupling matrix reveals the system's supermodes, and from these modes, we calculate the localisation plotted in the main text. In the main text, the three systems where we compute this localisation are: (1) a topological edge mode in the fabricated fibre model, (2) an initially delocalised mode in the ring fibre, and (3) a trivially localised edge mode in the 1700 rad/m twist fibre.

To quantitatively assess the effect of disorder on the supported supermodes, we calculate an intensity difference between an ideal edge mode (where the intensity is uniformly distributed across all cores) and the supermodes supported by each fibre model. We take the difference in intensity at each edge core, square the values so that positive and negative changes do not cancel out, and sum over all of these residuals corresponding to the squared intensity difference. In the main text, we dub this value the Edge Core Intensity Difference, and the modes with the lowest value are the closest to an ideal edge mode. We first use this metric to find the most uniform edge modes in each system for the case with no disorder (corresponding to the most ideal edge state) and plot how these modes' Edge Core Intensity Difference changes as a function of disorder (shown in Fig. S5). To ensure we are seeing general effects, we consider 1000 realisations of 10 disorder strengths.

During fibre fabrication, fluctuations in parameters (such as temperature) during the drawing process can introduce disorder into any core in the cross-section. Due to the robust nature of topological edge modes, their supported

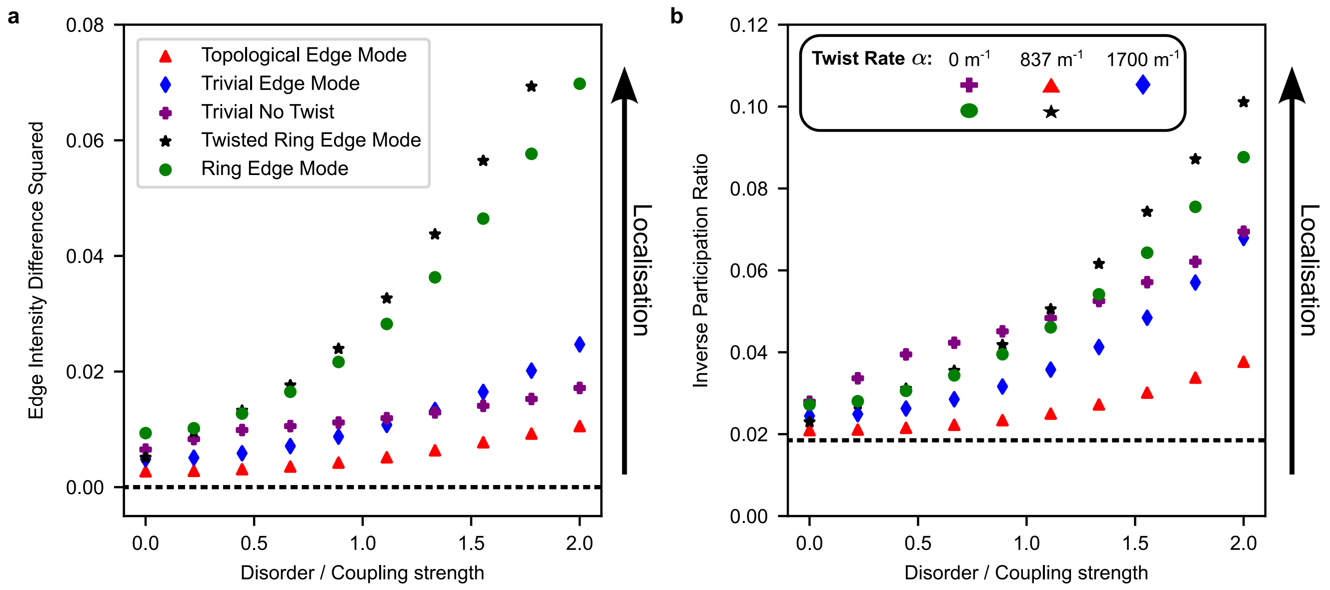

Fig. S5. Disorder robustness of different fibre cross-sections. We quantify the disorder localisation that occurs in a fibre cross-section when cores change size or shape and plot this for five different fibre cross-section geometries. **a**, The Edge Core Intensity Difference is calculated by first finding the intensity difference between a ideal edge mode (equal intensity in all edge cores) and the edge cores of each mode, then squaring and summing these differences to compute a single value. For each fibre cross-section, we compare the mode with the lowest edge core intensity difference in the presence of no disorder. **b** Same simulation data analysed by computing the Inverse Participation Ratio as a measure of disorder, which leads to the same conclusions as in **a**. We see that compared to all other fibre structures, the topological edge mode remains the most delocalised (and therefore, closest to an ideal edge mode) across all disorder strengths.

propagation constants change less than trivial bulk modes when exposed to disorder in the lattice. To explore this, we start with the density of states of our model fibre, and introduce random disorder along the diagonal of the coupling matrix. We draw random values from a uniform distribution between $\pm 1/2$. We then multiply these values by the disorder strength and add these values to the diagonal of the coupling matrix. Once the new coupling matrix is defined, we calculate the density of states. This is repeated and averaged over 2000 iterations to show both the average change in the density of states and their standard deviations. As we report in the main text, for small disorder, the standard deviation in the density of states of the topological edge modes (red and blue) is much smaller than the deviations present in the bulk modes (black). As the magnitude of disorder is increased up to the coupling strength C, we see that the topological protection of the edge modes begins to break down and the standard deviation of the density of states becomes comparable to that of the bulk modes.

In the main text we focus on the introducing disorder into the diagonal of the coupling matrix. We specifically look at the on-site terms because previous topological fibre did not protect against disorders to individual cores. However, the robustness our fibre demonstrates is not limited to the on-site terms. For systems characterised by Chern numbers, the topological protection has no symmetry constraints, and as such, any disorders introduced to the coupling matrix are protected against. We verify this in Fig. S5 where we introduce disorder into all terms in the coupling matrix (Fig. S5**a**,**b**) and the off-diagonal terms separately (Fig. S5**c**,**d**). We show that similarly to the results in the main text, the topological edge mode changes the least in the presence of introduced disorder.

IV. SUPPLEMENTARY VIDEO CAPTIONS

Supplementary Video 1. **Simulated intensity transport in the fibre cross-section using the tight-binding model.** Left: Light coupled into the topological fibre in the $\mathcal{C} = 1$ edge mode propagates anti-clockwise. Middle: Coupling into the $\mathcal{C} = -1$ mode yields clockwise propagation. Right: In an untwisted, topologically trivial fibre, the same initial excitation as the $\mathcal{C} = 1$ case does not show chiral motion.

Supplementary Video 2. **Chiral intensity transport and twist direction.** Left: In an anti-clockwise-twisted fibre, a $\mathcal{C} = -1$ mode propagates clockwise. Middle: In a clockwise-twisted fibre, the same mode propagates anti-

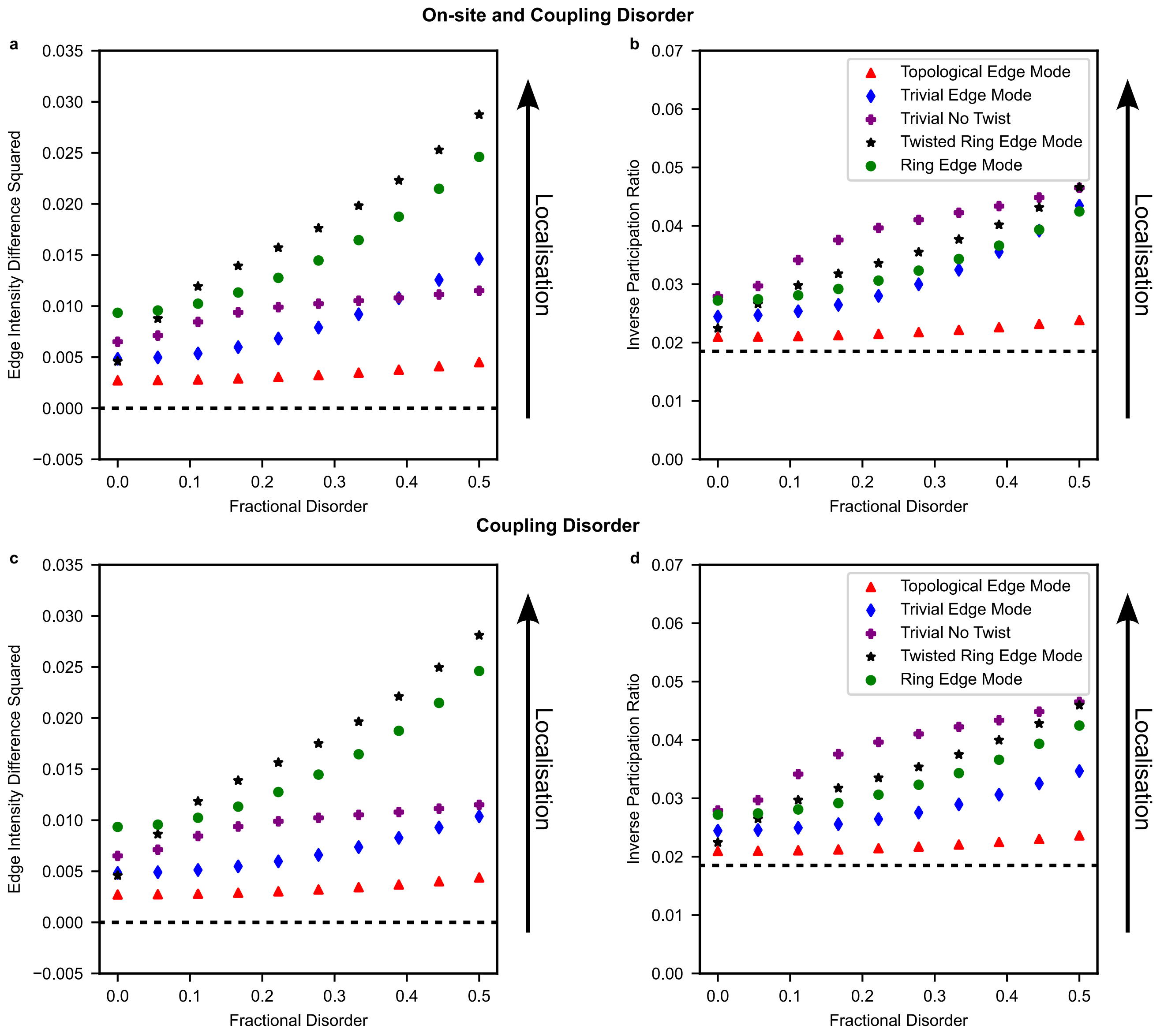

Fig. S6. Disorder robustness of different fibre cross-sections when the disorder is introduced in the coupling strengths. We quantify the disorder localisation that occurs in a fibre cross-section when cores change location (Coupling Disorder) and when cores change location and size/shape (On-site and Coupling Disorder). We plot this for five different fibre geometries. **a**, We use the Edge Core Intensity Difference (defined in main text and Fig. S5), to compare the localising effect of disorder in each fibre cross-section. For each cross-section, we compare the mode with the lowest edge core intensity difference in the presence of no disorder. To introduce disorder, we scale both the on-site and coupling terms by random fractions (derived by drawing a value from a uniform distribution between -0.5 and 0.5, multiplying this value by "Fractional Disorder", then adding 1). We ensure the same cores/couplings are introduced to the same disorders across all models. **b** Same simulation data analysed by computing the Inverse Participation Ratio as a measure of disorder, which leads to the same conclusions as in **a**. We see that compared to all other fibre structures, the topological edge mode remains the most delocalised (and therefore, closest to an ideal edge mode) across all disorder strengths. **c**, We plot the localising effect of disorder in the coupling strength. As in **a**, we plot the Edge Intensity Difference Squared and show that the topological edge mode remains the least localised in the presence of coupling strength disorder. **d**, Computing the Inverse Participation Ration reveals the same trend as **c**. The localising effect of disorder (in the coupling strength between cores) is reduced in the topological edge modes.

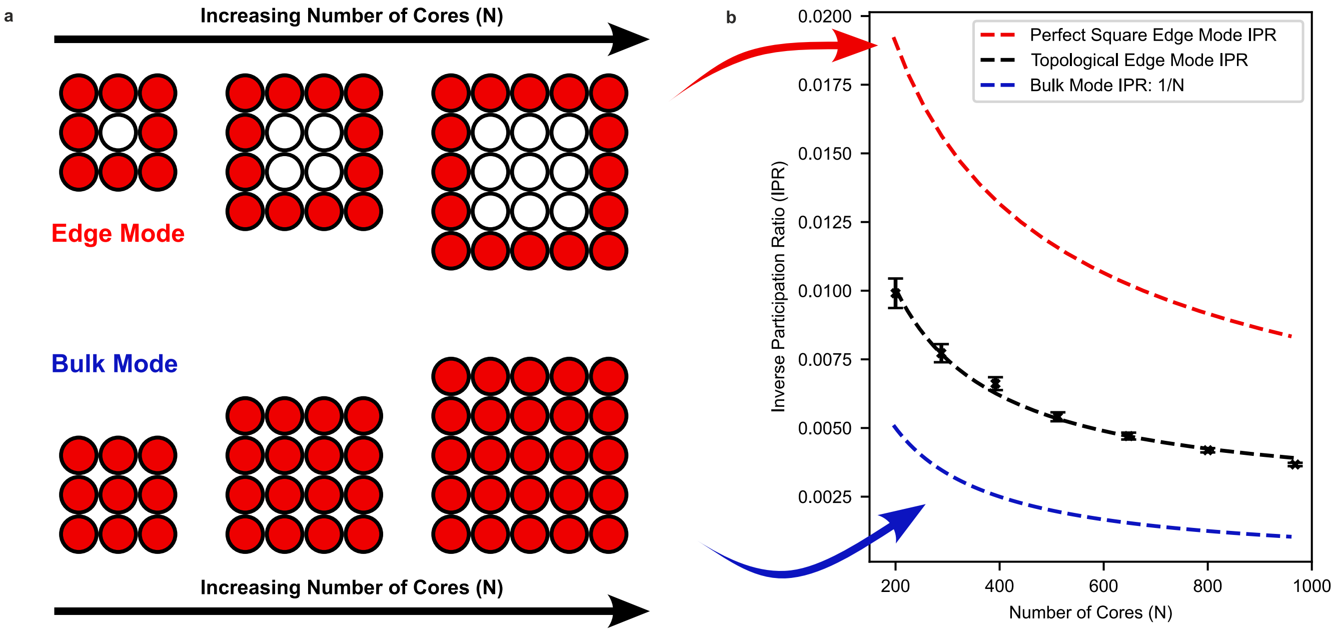

Fig. S7. Inverse participation ratio (IPR) and system size scaling. **a**, Schematic diagram of edge-localised and delocalised modes as a function of system size. **b**, Inverse participation ratio dependence on system size for three types of mode: Perfect square edge mode (top schematic in **a**), Totally delocalised bulk mode (bottom schematic in **a**), Topological mode from $\mathcal{C} = -1$ band gap, when no centripetal potential is considered. Black crosses correspond to tight-binding calculated values, error bars show the standard error, and the dashed black line shows the fitted data ($IPR = 1.55/N$). To calculate the average IPR of a topological mode as a function of system size, a honeycomb lattice is masked with a variably sized rhombus to keep a consistent shape while changing the number of cores. To avoid mode-specific effects, we average over 5 modes within the band gap. The IPR calculated for modes within the $\mathcal{C} = -1$ band gap remains significantly greater than the expected for bulk-scaling modes, further confirming their existence as topological edge modes.

clockwise. Right: In an untwisted, trivial fibre, the excitation does not exhibit chiral motion.

Supplementary Video 3. **Finite-element simulation of light propagation in our fibre model.** Left: A selection of an E_x polarised $\mathcal{C} = -1$ mode is used as an initial excitation in a finite-element simulation. This initial profile excites a fibre with a twist rate of 837 rad/m. The intensity moves clockwise around the fibre cross-section while it propagates along the length of the fibre. Right: The same initial excitation propagates in an untwisted trivial fibre. There is no chiral transport of light intensity in the cross-section.

Supplementary Video 4. **Circularly polarised light propagation in our fibre model (finite-element simulation).** Left: Half of a left-circularly polarised $\mathcal{C} = -1$ mode is used as an intial excitation in a finite-element simulation. This initial profile excites a fibre with a twist rate of 837 rad/m. The intensity moves clockwise around the fibre cross-section while it propagates along the length of the fibre. Right: Half of a left-circularly polarised $\mathcal{C} = +1$ mode is used as an initial excitation. This initial profile excites a fibre with a twist rate of 837 rad/m. The intensity moves anti-clockwise around the fibre cross-section while it propagates along the length of the fibre.

Supplementary Video 5. **Chiral transport for different twist rates (tight-binding model).** Left: A fibre twisted at 200 rad/m is excited using a mode with a real-space Chern marker of $\mathcal{C} = -0.47$. Clockwise edge transport is present but weak due to significant bulk overlap. Middle: At 400 rad/m, excitation using a $\mathcal{C} = -0.74$ shows clearer clockwise chiral transport, with reduced reduced bulk overlap. Right: At 600 rad/m, excitation using a $\mathcal{C} = -0.8$ mode shows strong clockwise chiral transport and minimal bulk overlap.

[1] Rechtsman, M. C. *et al.* Photonic Floquet topological insulators. *Nature* **496**, 196–200 (2013).
[2] Ross, J. The rotation of the polarization in low birefringence monomode optical fibres due to geometric effects. *Optical and Quantum electronics* **16**, 455–461 (1984).
[3] Bliokh, K. Y. Geometrodynamics of polarized light: Berry phase and spin hall effect in a gradient-index medium. *Journal of Optics A: Pure and Applied Optics* **11**, 094009 (2009).
[4] Sakurai, J. J. *Modern Quantum Mechanics* (Benjamin/Cummings, 1994).

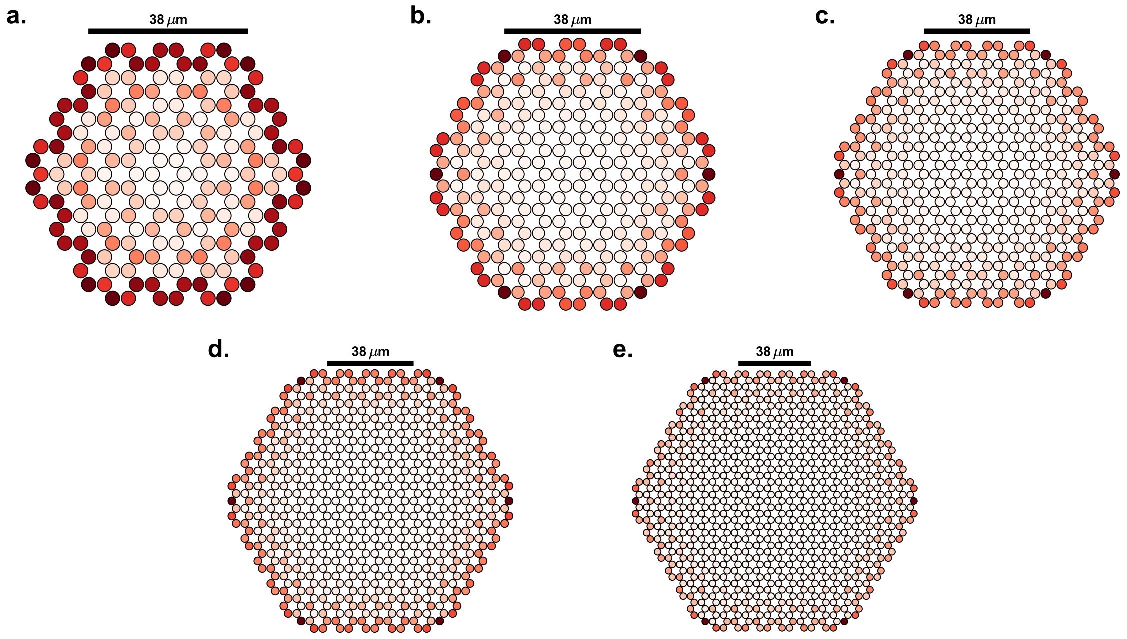

Fig. S8. Increasing the number of cores in the fibre (while keeping all other parameters fixed) leads to a larger lattice and overall fibre diameter. A larger diameter increases the deleterious centripetal term, but by reducing the twist rate appropriately as the lattice scales, topological edge states can be preserved. **a**, Topological edge state in a fibre with 180 cores, twisted at 800 rad/m. **b**, Topological edge state in a fibre with 258 cores, twisted at 550 rad/m, **c**, Topological edge state in a fibre with 414 cores, twisted at 350 rad/m, **d**, Topological edge state in a fibre with 606 cores, twisted at 350 rad/m, **e**, Topological edge state in a fibre with 834 cores, twisted at 200 rad/m.

[5] Kishi, N. & Yamashita, E. A simple coupled-mode analysis method for multiple-core optical fiber and coupled dielectric waveguide structures. In *1988., IEEE MTT-S International Microwave Symposium Digest*, 739–742 vol.2 (1988).

[6] Russell, P. S., Beravat, R. & Wong, G. K. Helically twisted photonic crystal fibres. *Philosophical Transactions of the Royal Society A: Mathematical, Physical and Engineering Sciences* **375**, 20150440 (2017).

[7] Peierls, R. Zur theorie des diamagnetismus von leitungselektronen. *Zeitschrift für Physik* **80**, 763–791 (1933).

[8] Luttinger, J. M. The effect of a magnetic field on electrons in a periodic potential. *Phys. Rev.* **84**, 814–817 (1951).

[9] Fang, K., Yu, Z. & Fan, S. Realizing effective magnetic field for photons by controlling the phase of dynamic modulation. *Nature Photonics* **6**, 782–787 (2012).

[10] Lado, J., García-Martínez, N. & Fernández-Rossier, J. Edge states in graphene-like systems. *Synthetic Metals* **210**, 56–67 (2015).

[11] Souslov, A., Dasbiswas, K., Fruchart, M., Vaikuntanathan, S. & Vitelli, V. Topological waves in fluids with odd viscosity. *Phys. Rev. Lett.* **122**, 128001 (2019).

[12] Kitaev, A. Anyons in an exactly solved model and beyond. *Annals of Physics* **321**, 2–111 (2006). January Special Issue.

[13] Mitchell, N. P., Nash, L. M., Hexner, D., Turner, A. M. & Irvine, W. T. Amorphous topological insulators constructed from random point sets. *Nature Physics* **14**, 380–385 (2018).

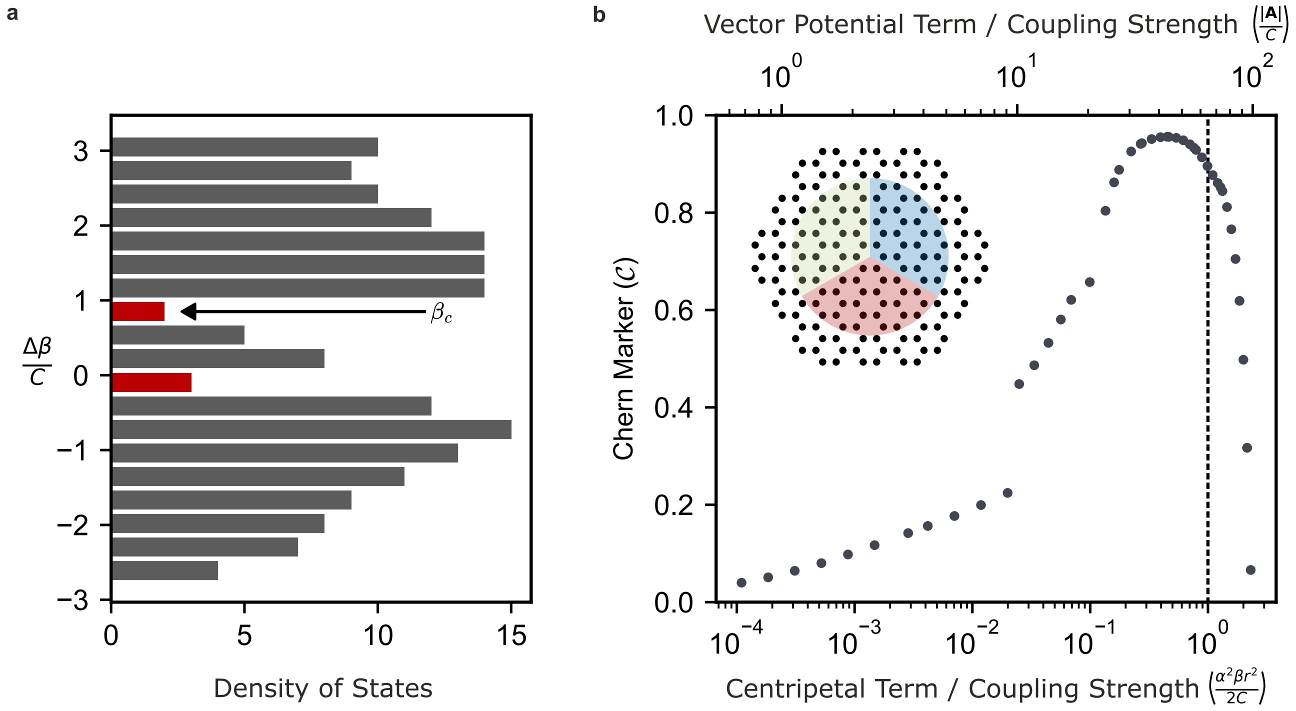

Fig. S9. Real-space Chern marker calculation and effect of twist-induced scalar potential. **a**, The propagation constant density of states is calculated using tight-binding numerics. We label the propagation constant cut-off β_c, which is used to compute the real-space Chern marker shown in **b**. **b**, Real-space Chern marker of the upper topological region (above the cutoff shown in **a**) is plotted as a function of twist, here non-dimensionalised in two ways: as ratios of the vector potential (top horizontal axis) and the centripetal scalar potential (bottom horizontal axis) and the coupling strength C (at zero twist). The graph shows that for both small twist rates and large twist rates, the fibre is topologically trivial, but for intermediate values of the twist rate, the Chern marker indicates non-trivial topological character. The real-space Chern marker in **b** and density of states in **a** have been calculated for the lattice shown in the inset of **b**.

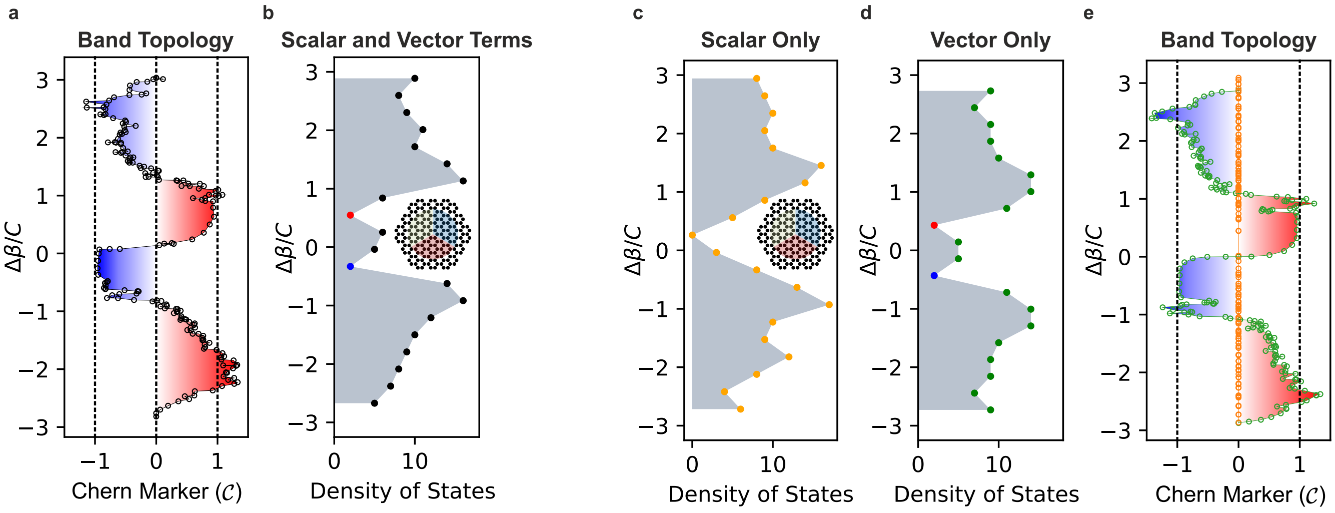

Fig. S10. Calculating the real-space Chern marker (which approximates the Chern number) for each gap present in the structure of supported propagation constants. **a**, The Chern marker (for the fibre cross-section shown in the inset of **b**) is plotted on the x-axis, with the associated propagation constant cutoff (used to calculate the Chern marker) on the y-axis. **b**, The density of states of the fibre's supported propagation constants, which shows the location of the two sparsely populated regions (topological gaps). The two gaps have been coloured to represent their respective Chern markers. Red corresponds to a Chern marker of 1 and blue corresponds to a Chern marker of -1. Both centripetal terms and vector potential terms are included in the calculation. **c**, Density of states for a twisted system when ignoring the effects of the vector potential. This system does not break time-reversal symmetry and exhibits only trivial Chern markers ($\mathcal{C} = 0$) for all propagation constant cut-off values, which are shown with orange symbols in **e**. **d**, Density of states for a twisted system with no centripetal terms, which does exhibit non-trivial Chern marker values. The calculated Chern markers ($\mathcal{C} \neq 0$) are shown in green in **e**. This figure uses fibre parameters corresponding to the twist rate of 866 rad/m, and a coupling strength C of 6182 m^{-1}.

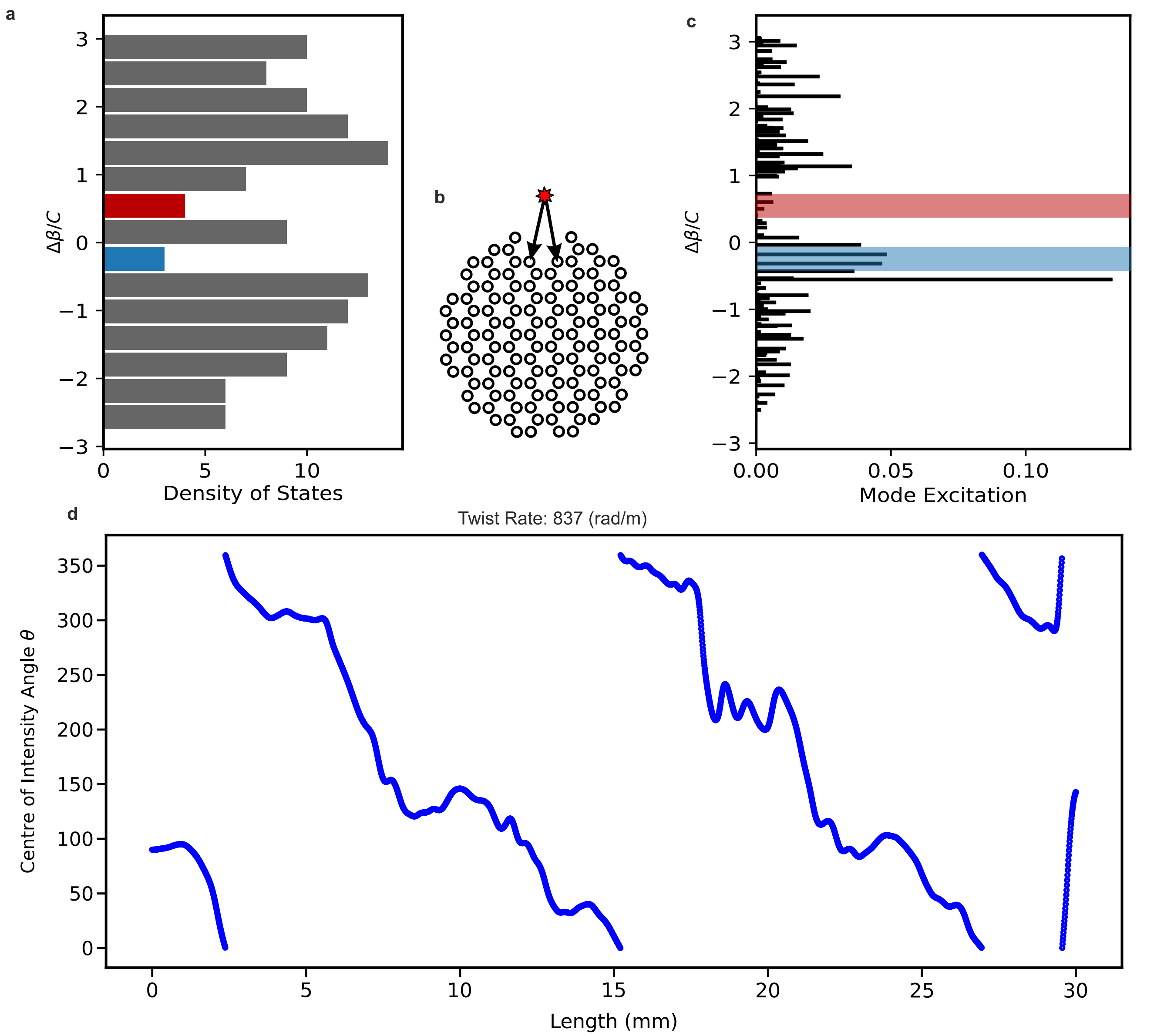

Fig. S11. Exciting $\mathcal{C} = -1$ states and observing chiral transport. **a**, The density of states is plotted for supported propagation constants. The model fibre mirrors the one used in the experimental data and is twisted at a rate of 837 rad/m. **b**, Geometry of the fibre model used in our analytic model and experiment. The red star indicates where light is injected to excite states in the $\mathcal{C} = -1$ (blue) gap. **c**, Overlap integrals between the supermodes (for each $\Delta\beta$) and the cores highlighted in **b**. Injecting into these two cores excites states in the lower gap region ($\mathcal{C} = -1$, blue) more than the counter-propagating mode ($\mathcal{C} = 1$, red). **d**, The centre of intensity is calculated and the angle it subtends is plotted as a function of propagation length. Light intensity propagates around the structure in the opposite direction to the $\mathcal{C} = 1$ gap states.

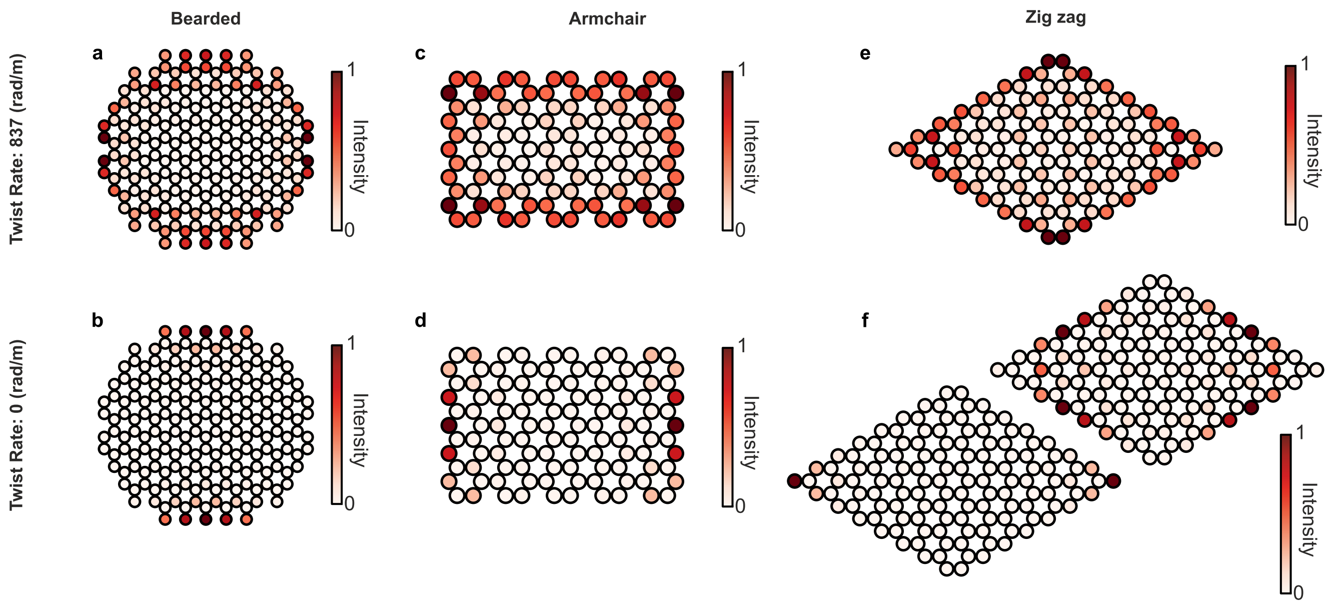

Fig. S12. Edge localised states in the trivial and topological regimes. The topological regime uses a model fibre that mirrors the one used in the experimental data and is twisted at a rate of 837 rad/m **a**, Intensity profile of a topological edge mode in a fibre geometry that features bearded edges. When the fibre is twisted intensity becomes localised across all edge cores of the structure. **b**, Intensity profile of a trivial mode that features localisation due to the bearded edges. When the fibre is untwisted the geometry gives rise to edge-dependent localised modes that live on the bearded edges and are not topologically protected. **c**, Intensity profile of a topological edge mode in a fibre geometry that features armchair edges. As in **a**, once the fibre is twisted intensity becomes localised across all of the edge cores. **d**, Intensity profile of a trivial mode that features no localisation on the armchair edges, but as expected supports edge states on the zigzag edges. **e**, Intensity profile of a topological edge mode in a fibre geometry that features zigzag edges. As in **a**, once the fibre is twisted intensity becomes localised across all of the edge cores. **f**, Two intensity profiles of trivial modes featuring edge localisation are plotted. The zigzag edges support both single-core and multi-core edge states in the topologically trivial (0 rad/m twist rate) regime.

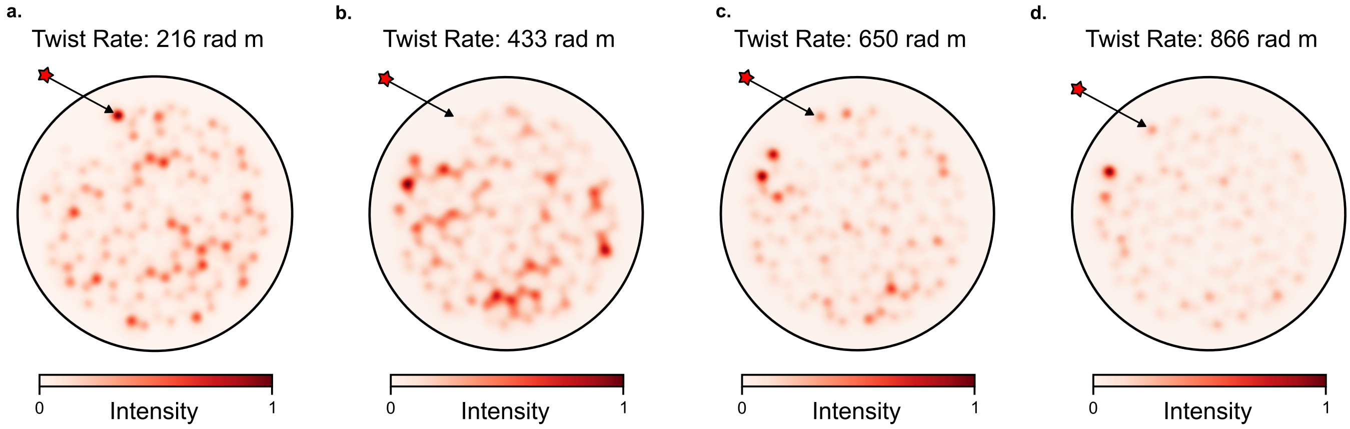

Fig. S13. Varying twist rate in experimental results. Light propagates through four different fibres, fabricated with different twist rates but the same cross-sectional structure. Light is injected into the marked core and propagates through (23 ± 2) mm of each fibre. We see increasing localisation of intensity as a function of the fibre twist rate. **a**, Light is injected into a lightly twisted fibre (216 rad/m) and is not localised to the perimeter. **b**, Light propagates through a fibre with a twist rate 433 rad/m and remains delocalised at the output. **c**, Intensity becomes more localised at the edge of the lattice when light propagates through a fibre with twist rate 650 rad/m. **d**, Edge-localisation of intensity can be seen when light propagates through a fibre with twist rate of 866 rad/m.

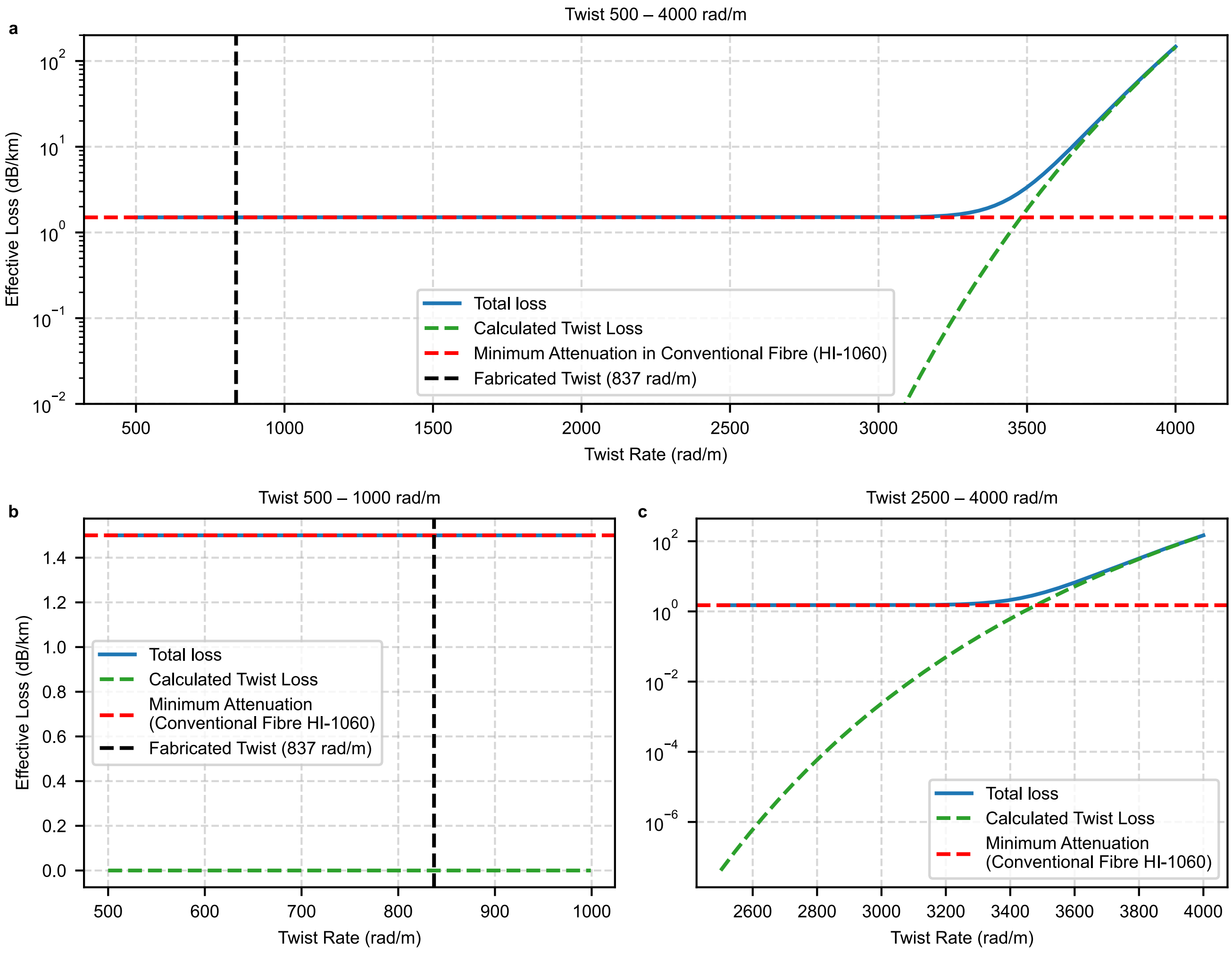

Fig. S14. Effective bend loss induced by twisting. Twisting the fibre introduces a curvature that could give rise to excess loss. We model this additional loss and compare it to the attenuation in conventional fibre. **a**, Effective loss is plotted over a range of twist rates 500 – 4000 $\mathrm{rad\,m^{-1}}$. The blue line shows combined material and curvature loss. The green dashed line corresponds to the additional loss due to twisting. We model the loss due to twisting by calculating the bend loss an identical fibre, with equivalent curvature, experiences. The red dashed line shows the minimum attenuation of conventional fibre at 1 µm. The black dashed line shows the twist rate of our fabricated fibre. **b**, Effective bend loss induced by twisting between 500 – 1000 $\mathrm{rad\,m^{-1}}$. **c**, Effective bend loss induced by twisting at higher twist rates (2500 – 4000 $\mathrm{rad\,m^{-1}}$)

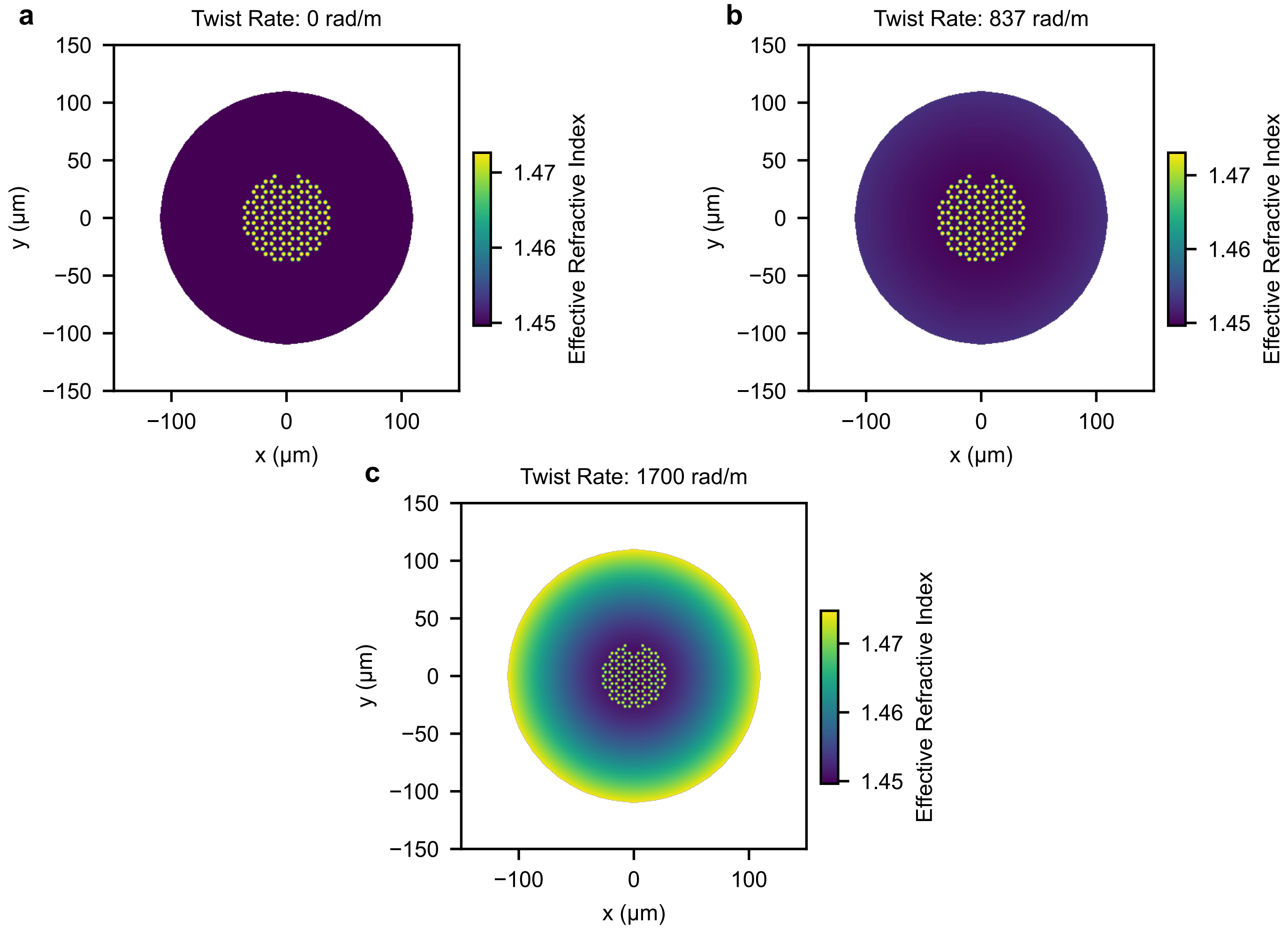

Fig. S15. Refractive index distribution in the fibre cross-section for three different twist rates. a, The untwisted refractive index distribution. b, Effective refractive index distribution when the fibre is twisted with a twist rate 837 rad/m (our fabricated fibre). c, Effective refractive index distribution when the fibre is twisted with a twist rate of 1700 rad/m.
